# Novel way to investigate evolution of children refractory epilepsy by complexity metrics in massive information

**DOI:** 10.1186/s40064-015-1173-6

**Published:** 2015-08-21

**Authors:** Ricardo Zavala-Yoé, Ricardo Ramírez-Mendoza, Luz M Cordero

**Affiliations:** Tecnológico de Monterrey, Escuela de Ingeniería y Ciencias, Calle del Puente 222, Col. Ejidos de Huipulco, 14380 México DF, Mexico City, Mexico; Instituto Nacional de Pediatría, Av. Insurgentes Sur 3700 C, Coyoacán, Insurgentes Cuicuilco, 04530 Mexico City, Mexico

**Keywords:** Complexity, Epilepsy, Doose Syndrome, Multiple long term EEG

## Abstract

Epilepsy demands a major burden at global levels. Worldwide, about 1% of people suffer epilepsy and 30% of them (0.3%) are anticonvulsants resistant. Among them, some children epilepsies are peculiarly difficult to deal with as Doose syndrome (DS). Doose syndrome is a very complicated type of *children cryptogenic refractory epilepsy* (CCRE) which is traditionally studied by analysis of complex electrencephalograms (EEG) by neurologists. CCRE are affections which evolve in a course of many years and customarily, questions such as on which year was the kid healthiest (less seizures) and on which region of the brain (channel) the affection has been progressing more negatively are very difficult or even impossible to answer as a result of the quantity of EEG recorded through the patient’s life. These questions can now be answered by the application of entropies to massive information contained in many EEG. CCRE can not always be cured and have not been investigated from a mathematical viewpoint as far as we are concerned. In this work, a set of 80 time series (distributed equally in four yearly recorded EEG) is studied in order to support pediatrician neurologists to understand better the evolution of this syndrome in the long term. Our contribution is to support multichannel long term analysis of CCRE by observing simple entropy plots instead of studying long rolls of traditional EEG graphs. A comparative analysis among aproximate entropy, sample entropy, our versions of multiscale entropy (MSE) and composite multiscale entropy revealed that our refined MSE was the most convenient complexity measure to describe DS. Additionally, a new entropy parameter is proposed and is referred to as bivariate MSE (BMSE). Such BMSE will provide graphical information in much longer term than MSE.

## Background

### Introduction

Epilepsy has to be determined by several diagnostic tests. One of the them is the electroencephalogram (EEG). Frequently, this is the first test chosen by neurologists and when a patient is diagnosed to suffer such disease it is necessary to practice several EEG studies yearly. As time goes by, the accumulation of EEG studies implies a massive storage of information, specially when the epilepsy results difficult to deal with. Epilepsy demands a major burden at global levels. Worldwide, about 1% of people suffer epilepsy and 30% of them (0.3%) are anticonvulsants resistant. Among them, some children epilepsies are peculiarly difficult to deal with. Not only for being antiseizure medications resistant but also for being time varying. Childhood syndromes are typical cases of this. Nonlinear analysis of electroencephalographic signals can help to understand better a very difficult case of abnormal dynamics in brain, the Doose Syndrome (DS) (Stephani 2006; Doege et al. 2014; Von Spiczak et al. 2011; Kelley 2010). DS is a type of children epilepsy described by serious alterations in the EEG. However, sometimes it is difficult to give a definitive diagnostic of such disease because of overlapping with other similar pathologies. Moreover, this affection may evolve to other types of epilepsy complicating more a differential diagnostic. In addition, the aetiology (cause) of this class of illness is sometimes unknown. In this case, the type of epilepsy is referred to as *cryptogenic*. As a result, anticonvulsants do not cure the ill and so the epilepsy is known as *refractory*. The case of DS considered in this work belongs the this class of combined characteristics and is known as *cryptogenic refractory children epilepsy* (CRCE). Moreover, this affection is multifocal and may evolve to other types of epilepsy complicating more a differential diagnosis. When a pediatric neurologist wants to diagnose DS in a child, his/her first step is to analyze a set of EEG. This set may consist of many volumes of EEG studies accumulated during the patient’s history. However, recording an EEG typically lasts one or more hours which implies a lot of printed paper with long-term plots. Since the development of the affection must be studied, the neurologist has to compare several EEG among them at the same time. But in addition, these EEG are plenty of abnormalities (high frequency spikes, polyspikes, slow waves, etc.) all of this resulting in an unhandy volume of information. In this sense, the contribution of this paper is twofold: First, a bidimensional (2D) graphical alternative to understand in a much simpler way than before the evolution of this DS is given in terms of the *complexity * of each time series. Comparing long term EEG-changes through different years is now easier by analyzing complexity plots than proceeding as usual. Such graphs are interpreted here from a mathematical-medical point of view. As a result of our contribution, neurologists can be supported by math modelers in order to understand better the progress of the affection or even to find a cure for a very serious disease. The idea offered in this work can be extended to more types of CCRE. Second, an additional contribution of this work is to provide MATLAB-based-3D-graphical criteria (in terms of BMSE) to describe and understand better the long term evolution of DS. In this sense, since typical one-page plots of EEG (known as epochs) last typically 10 s, *long term* will be understood here as a period which ranges from some minutes to several hours, i.e., periods which are not visible in only one EEG page or even in a complete EEG study. It will be shown later that periods of study of hours can be clearer exhibited in static or dynamic 3D graphs by means of BMSE. So, SBMSE and DBMSE will stand for static and dynamic BMSE, respectively. BMSE will be very useful in a situation like this: Think for instance in a set of EEG recorded two or three times a year during 3, 4, 5 or more years to study the evolution of a child’s epilepsy. And moreover, consider as well to compare those periods with another child (or children) EEG. Since the volume of information becomes unhandy and not comparable, hence subjectivity tends to increase.

As a further matter, there is no gold standard for an EEG’s true interpretation (Rating 2014; Grant et al. 2014). It is a rather subjective and experience-based activity. In “3D-MSE: BMSE numerical experiments as clinical interpretation” it will be shown that this intuitive character in EEG’s interpretation can be assisted by the use of BMSE. Note 1 about nomenclature. BMSE can be used generically for SBMSE and DBMSE.

As far as we are concerned, our contribution is new in CCRE and DS. Hopefully, better treatments to cure DS can be explored in this way.

### Some issues about epilepsy

**Fig. 1:**
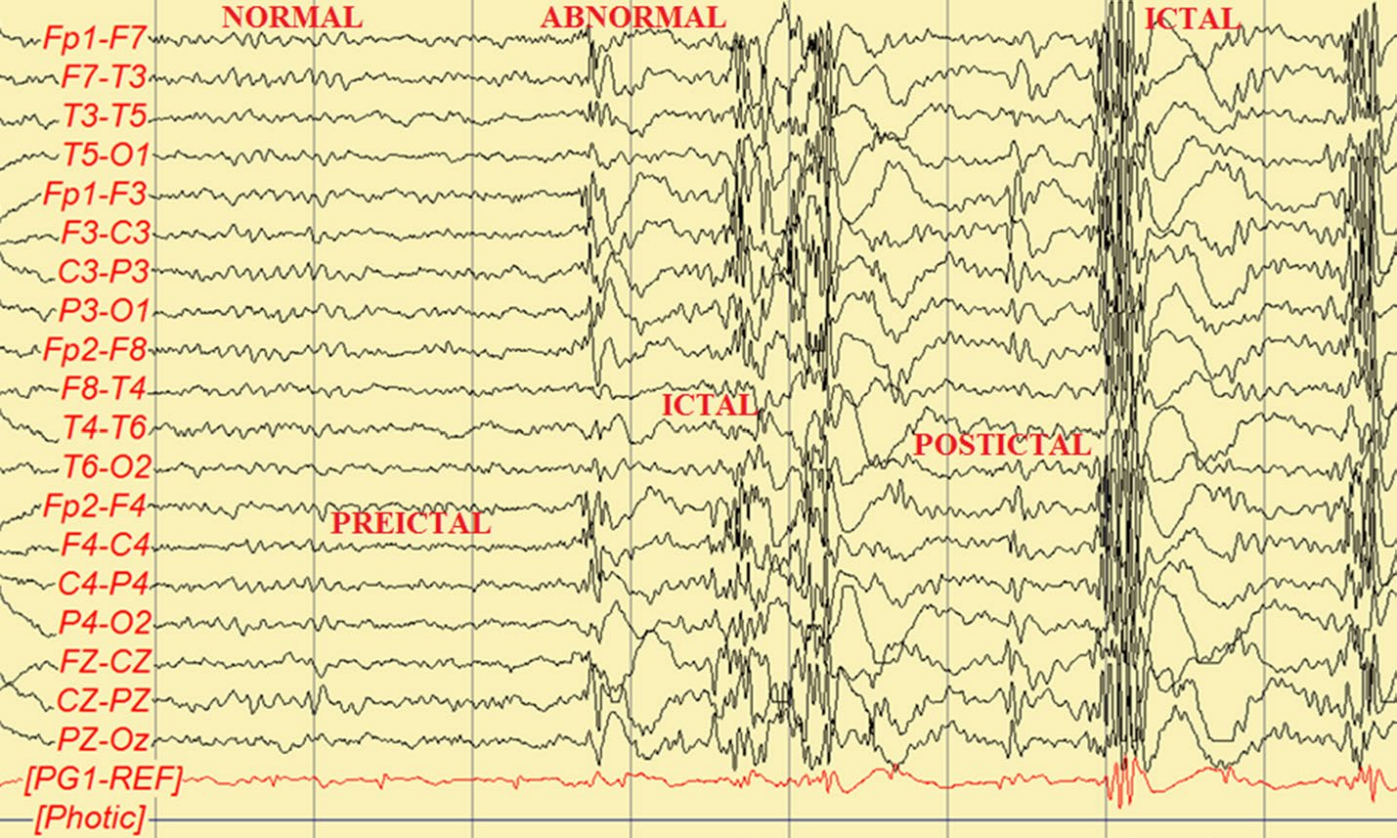
Normal and abnormal findings in a DS EEG. Bursts of spike-wave activity superimposed on an otherwise normal background. Preictal, ictal and postictal stages are also indicated.

International League Against Epilepsy (ILAE) defines the following terms (Shorvon 2011). An epileptic seizure is a transient occurrence of signs and/or symptoms due to abnormal excessive or synchronous neuronal activity in the brain. Normally, brain behavior is non-synchronous (Fig. 1). An epileptic seizure can last from a few seconds to more than five minutes at which point it is known as status epilepticus. Epilepsy is defined as a disorder of the brain characterized by an enduring predisposition to generate epileptic seizures, and by the neurobiological, cognitive, psychological, and social consequences of this condition. The definition of epilepsy requires the occurrence of at least one epileptic seizure (Shorvon 2011). The seizure stages are four: (a) Pre-ictal, which refers to the state immediately before the actual seizure; (b) Ictal, relates an state when actually the seizure appears; (c) Postictal applies to the state shortly after the event; (d) Interictal, means the period between seizures (Shorvon 2011; Stephani 2006; Gil-Nagel 2001). Note: The epileptic seizures stages considered in our study are: preictal, ictal and postictal. No interictal state was taken into account because this state resulted of relative low voltage with respect to the other three phases and do not give important information for our goals.

It is noteworthy mention that the aetiology-based classification of epilepsy is the following:

- *Idiopathic* Epilepsy of not known cause but presumably genetic.
- *Symptomatic* Epilepsy secondary to a condition affecting the brain.
- *Cryptogenic* Epilepsy of unknown origin but most likely to be symptomatic.

Temporary substitutions have been recently proposed for those terms as genetic, structural-metabolic, and unknown, respectively, but their use is still being discussed (Shorvon 2011; Wilmshurst et al. 2014).

#### Doose syndrome

A syndrome is a group of signs and symptoms that, added together, suggest a particular medical condition. The german doctor, Hermann Doose first described the features of a previously incompletely defined epilepsy syndrome characterized by very different seizures, consisting of jerks, sudden falls to the ground (drop attacks), or sometimes a jerk followed by a fall. Absence seizures can happen (when consciousness is lost briefly) as well as the so called generalized tonic-clonic seizures (stiffness and jerking of the whole body). The EEG may be initially normal, but development of the disease will exhibit patterns of generalized spike and wave activity, 4–7 rhythms/s and bursts. Photoparoxysmal reaction may be observed in the EEG as well. In addition, DS is well known as being very refractory to be treated with many anticonvulsants and with alternative therapies. The boundary of DS with other close syndromes as Lenaux-Gastaut, Dravet, Pseudo Lenox,etc. is fuzzy (Doege et al. 2014; Stephani 2006; Von Spiczak et al. 2011; Shorvon 2011; Kelley 2010).

#### The case under study: an overlapped DS

The child considered in this paper suffers DS as a particular case of CCRE. However, this child’s affection is an event where DS has not been possible to confirm as a result of overlapping with other similar pathologies. Although there exist genetic/metabolic tests to differentiate DS from close pathologies [as Nieman-Pick disease, Dravet syndrome, lipofuscinosis (Von Spiczak et al. 2011)] such tests have resulted negative in this child although clinical evidence (as EEG interpretation by neurologists) indicates that this affection is very probably a DS. We remark that this situation complicates even more finding a reliable treatment. About twenty five different types of anticonvulsants have been probed in our child with a very poor success (see “Anticonvulsants information”). Hence, since a good diagnosis is missing, we have to look for any alternative which could give a clue about the cause and the way such DS changes. As explained in “EEG databases”, there are four EEG recorded in 2008, 2010, 2011, and 2013, one per year. The EEG were retrieved from an important children hospital in Mexico City, where neurologists have given up (until summer 2014) as a result of the complexity of the case.

Some invasive techniques have been considered (vague nerve stimulator and corpus callosotomy) but their efficiency varies (Doege et al. 2014; Wakai and Kotagal 2001; Montavont et al. 2007; As and Smyth 2014). In addition, callosotomy is not reliable as a result of the presence of multifocal sources (Doege et al. 2014; Kelley 2010). Besides, ketogenic diet (KD) had also been taken into account but it was discarded as a result of pancreatitis suffered by this kid (see “Anticonvulsants information” and Doege et al. 2014). Hence, some other anticonvulsants from the few which have not been used have to be considered yet. Genetic studies have been also conceived to discover the aetiology of this CCRE but even finding the cause of this CCRE, would not change the medication (there does not exist a genetic “glue” to repair damaged genes or exomes Von Spiczak et al. 2011). Another complication *ex medicina* is the gap between resource—equipped and resource poor hospitals in some countries. Advances in medical treatments have been improved in so—called developed nations but the gap between resource—equipped and resource developing countries is remarkable. For instance, the quantity of demanding children to be attended may impose months-long waiting lists for admissions, even for seriously affected epileptic patients. These facts worsen seizures as well and normally they are not considered as exacerbating factors (Mbuba et al. 2008; Wilmshurst et al. 2014 and references therein).

### Note about pathologies in adults

Although some works in epilepsy have been done (see for instance Stam and van Straaten 2013; Bai and et al. 2010 and references therein), papers which investigate children epilepsies by entropy measures are practically null. Main attention has been devoted to adults affections. Serious diseases as Alzheimer Disease (AD), Creutzfeldt-Jakob Disease (CJD), schizophrenia, Parkinson Disease (PD) an others have already been studied from a quantitative point of view (Stam et al. 1997; Stam 2003; Stam and van Straaten 2013; Abasolo et al. 2008; Labate et al. 2014, 2013; Ahmed and Mandic 2011). Since math analysis of EEG records is involved, such field is referred to as Quantitative EEG (QEEG) by some authors. Nevertheless, as we remarked in the Abstract we are focused in children epilepsies (CCRE) and our contribution is new as far as we are concerned.

### Contributions as goals

Although the goals of this work were already explained in the Abstract and Introduction sections, the following list provides in detail the contributions of this document. They are shown to be accomplished through the development of “Results and discussion”. See also “Mathematical-medical relation: anticonvulsants and BMSE”.

- To evaluate a set of four entropy measures in order to obtain only one that can be useful to describe multiple long term EEG of CCRE (see beginning paragraph in “2D-complexity: improved MSE” and “ApEn, SaEn–CMSE analysis”)
- To analyze the long term development of a case of CCRE, DS by observation of multichannel EEG complexity plots in an easier way than studying unhandy information, i.e., hundreds of traditional time-voltage printed plots (“Entropy of the three stages of a seizure–DBMSE: computational animation of 2.5 h of BMSE evolution in EEG4”).
- To know how seizures stages look like in terms of flat entropies (“Entropy of the three stages of a seizure”).
- To make comparable multiple long term information (too long rolls of traditional plots can not be compared to each other easily and without subjectivity by neurologists, see “Comparison of preictal, ictal and postictal phases in multiple EEG”).
- To answer the following questions also posed by the parents of any child in this situation: which area of the brain is the most affected by seizures through the four years of studies and moreover, how is the evolution of the disease by brain zone through all this time? On which year was the kid healthiest and why? Naturally, the answers will influence medications, therapies, home-cares, and so on (“Multiple long term EEG analysis”).
- To propose a bivariate complexity parameter referred to as Bivariate Multiscale Entropy, BMSE (“Description of comparisons: static and dynamic BMSE”).
  The following goals are shown to be fulfilled in “Comparison of BMSE and MSE in X19 of EEG2 (2010)” and “Conclusions”:
- To comprise information of the evolution of DS in BMSE plots instead of studying hundreds of EEG traditional printed plots.
- To remove the subjective interpretation of EEG due to neurologists experience.
- To consider the use of BMSE in any other kind of CCRE.
- To make comparable long term EEG (for even longer periods than those deemed by MSE entropies) by BMSE.

## Results and discussion

### 2D-complexity: improved MSE

As it is explained in “Methods” the entire EEG database was analyzed with the four complexity measures. ApEn, SaEn, and MSE revealed well short term activity but CMSE not (see Algorithms). ApEn, SaEn are variance sensible to the length of the data series but MSE and CMSE are not. Memory restrictions have to be taken into account with ApEn (self matches need to be evaluated) and CMSE (extra coarse graining process computed) but not with SaEn and MSE (see “ApEn, SaEn”—“MSE analysis”). So, our analysis yielded that MSE was the most convenient measure to work with as a result of shorter computing time and fidelity in reproducing long and short term brain activity. In “Entropy of the three stages of a seizure” and “Comparison of preictal, ictal and postictal phases in multiple EEG” the three seizure stages considered here are analyzed in terms of MSE complexity. In addition, in “Multiple long term EEG analysis” from all the set of EEG (80 time series) it is concluded that the brain region F3 is the most affected through all these years of study. It is also deduced that 2010 was the worst year for the kid as a result of the low complexity and high variance shown in the corresponding plots.

#### ApEn, SaEn

From the results obtained by ApEn and SaEn it was concluded that the complexity of all EEG is low during ictal stages. This conclusion is in line with another serious neurological diseases as explained in “Note about pathologies in adults”. Without reviewing traditional EEG plots obtained in the hospital, it can be concluded from MSE curves that unfortunately, this person suffers a continuous state of discharges (voltage levels oscillates from high to very high). However, ApEn and SaEn are very sensitive to the length of data. By developing some algebra, we also concluded that standard deviation of both quantities are inversely proportional to such length of data N. See also “Aproximate entropy, ApEn”. In spite of this, some authors still like to use modified versions of ApEn or SaEn as nonlinear statistics (Sharanreddy 2013). This pair of metrics was implemented by Algorithms 1 and 2, respectively (see “Methods”).

#### MSE analysis

**Fig. 2:**
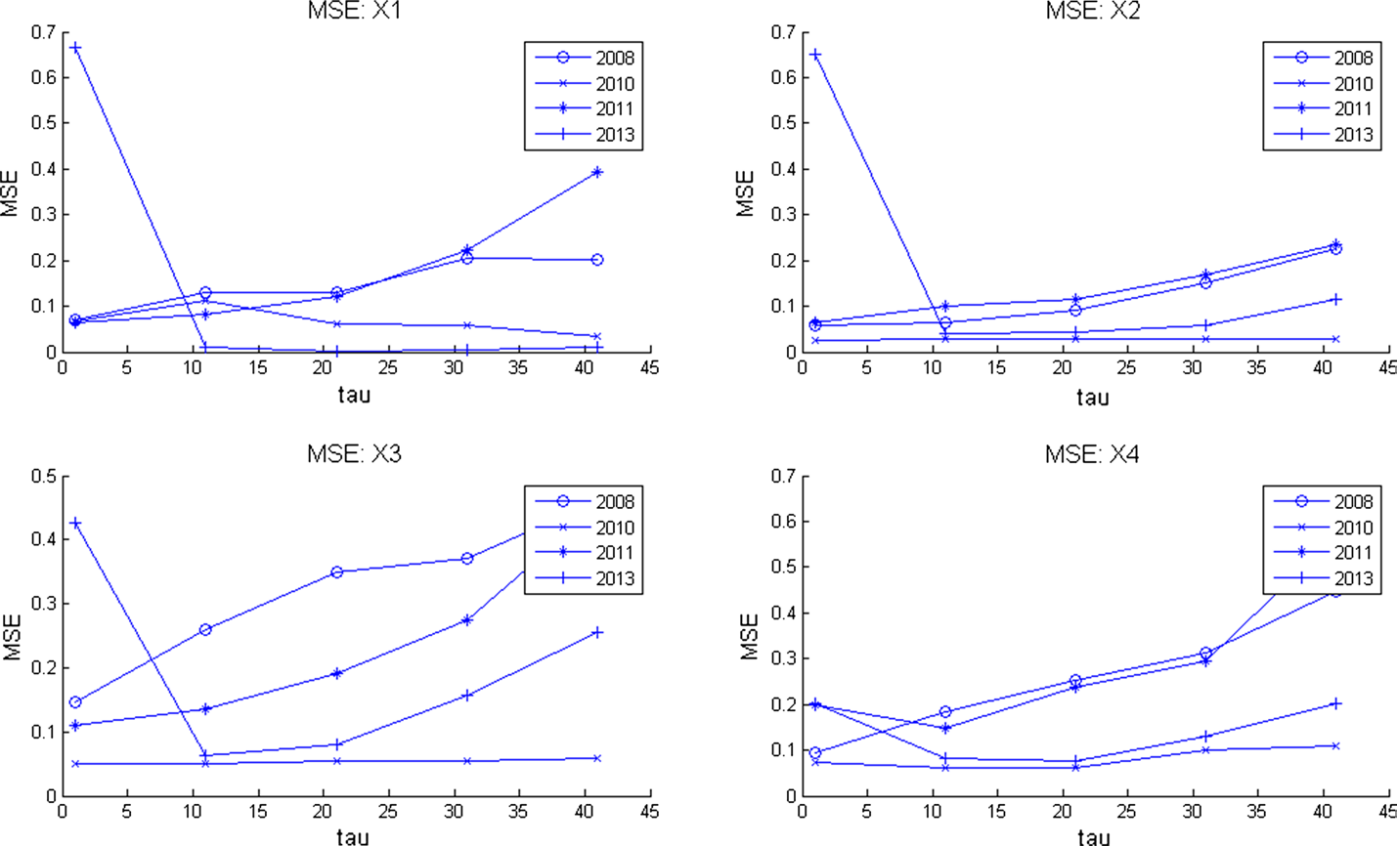
MSE during preictal phase in four channels during 5 years. The evolution of this DS is compared in four channels of EEG2. Observe how the general trend is upwards at this preictal stage.

**Fig. 3:**
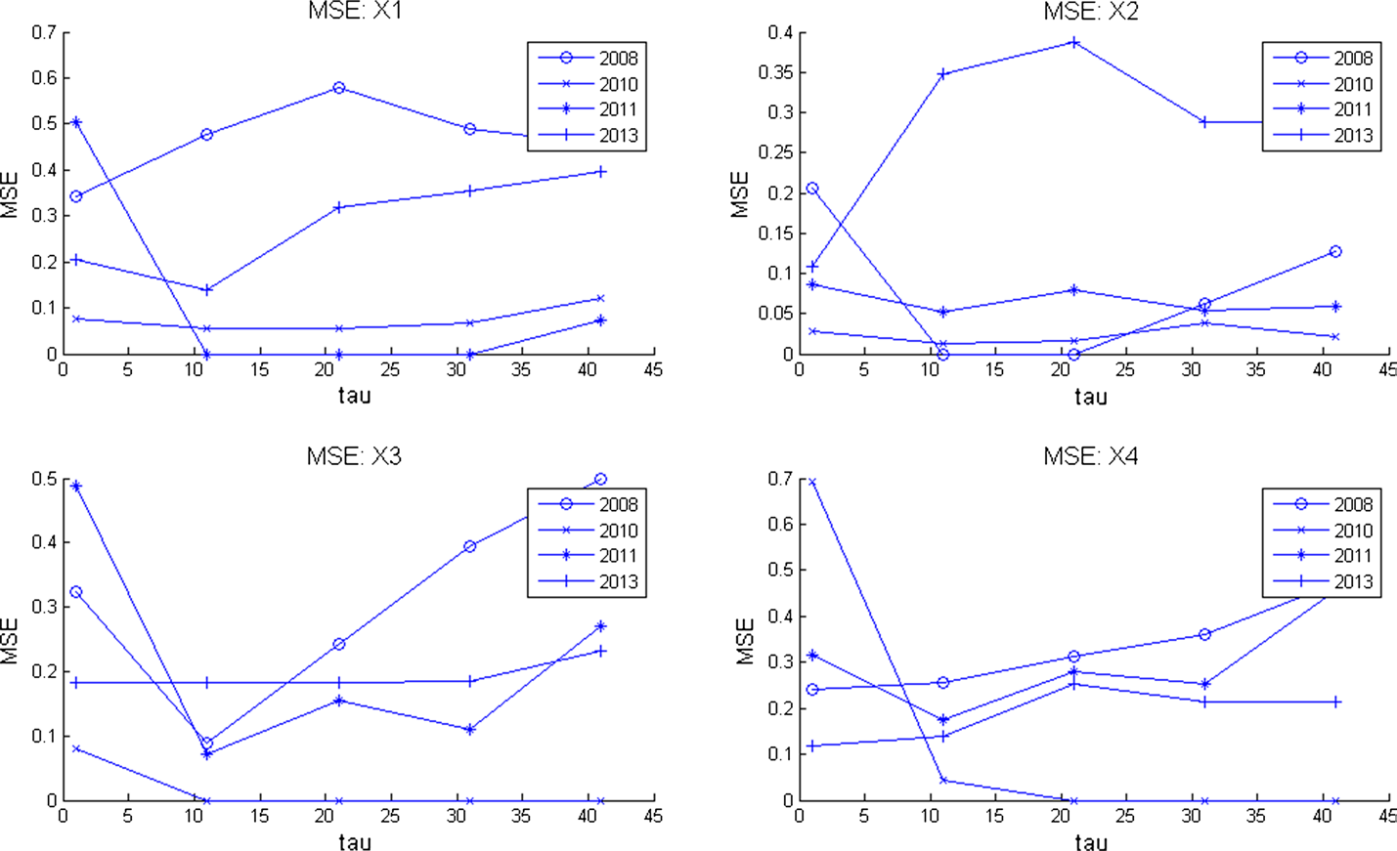
MSE during ictal phase in four channels during 5 years. The evolution of this DS is compared in four channels of EEG2. Observe how the general upwards trend of the preictal stage is distorted here.

**Fig. 4:**
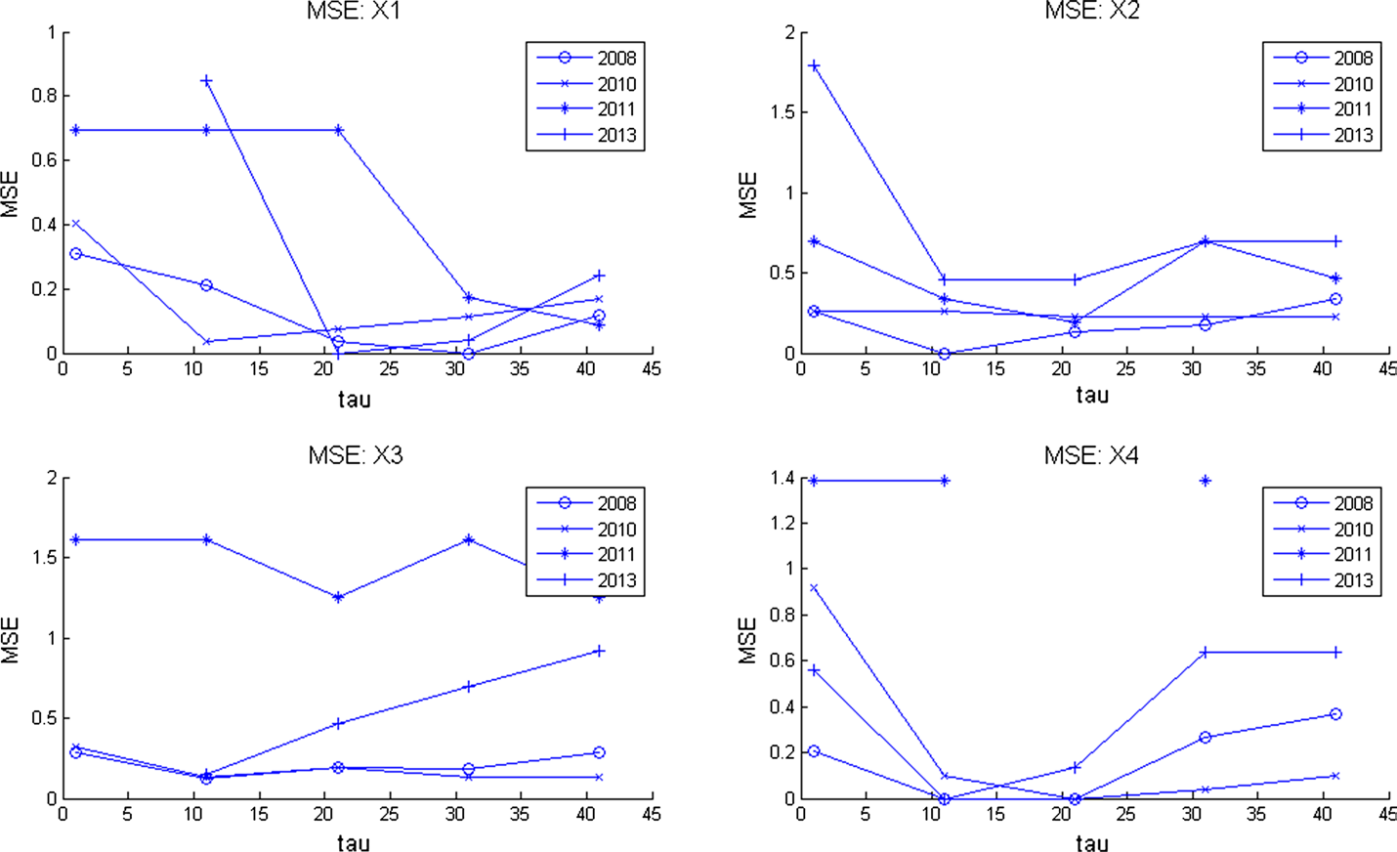
MSE during postictal phase in four channels during 5 years. The evolution of this DS is compared in four channels of EEG2 in a postictal stage. Notice how the how the general trend is upwards again, indicating that the brain recovers of the seizure.

**Fig. 5:**
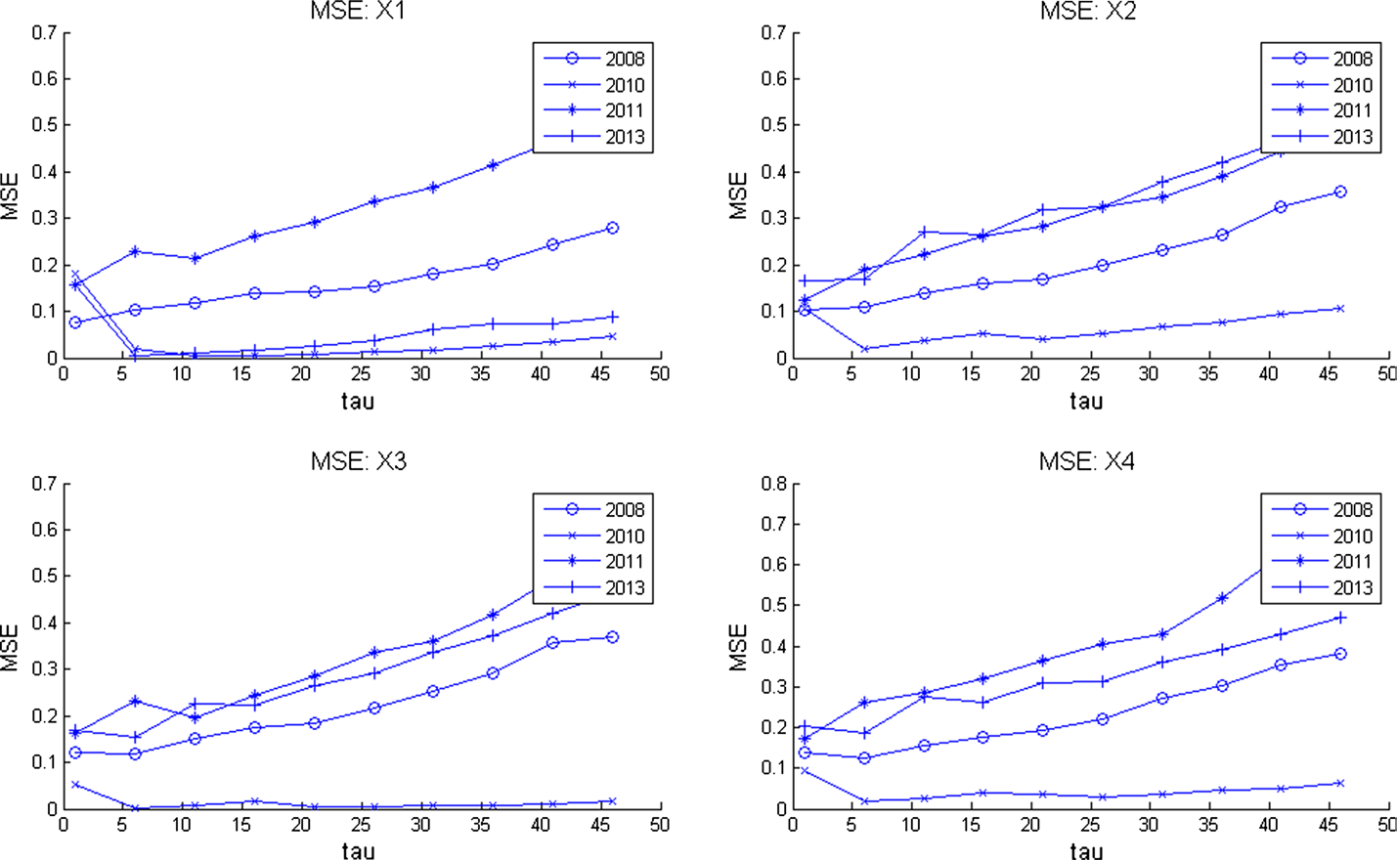
MSE behavior during 1 h time corresponding to one million samples through 5 years. This plot exhibits how this DS behaves in long term. The general trend is upwards, showing so that the child’s brain presents complex thoughts (playing, reading, walking, etc).

Computer memory restrictions are not that problematic as those presented during ApEn calculations. This is due to the fact that MSE starts from raw data (the original time series corresponding to an EEG channel) and later computes the set of coarse grained series for different time scales $\tau$. The computations are done in shorter and shorter time series (see Algorithm 3 in “Methods”) which converges to a faster computing time. This fact allowed to compute MSE for all channels during much longer EEG times. Broadly speaking, by observation of MSE plots (Figs. 2, 3, 4, 5) they indicate a relative low complexity in all EEG during states preponderantly ictal. This fact coincides with the conclusions obtained from ApEn, SaEn and CMSE (see “CMSE analysis”).

#### CMSE analysis

CMSE resulted to be too smooth in order to show fast variations of the EEG signals. This fact is explained by the extra coarse -graining process used in this computation (see Algorithm 4 in “Methods”). In addition, time calculation was also long with respect to the other entropies. Thus, the parameter chosen to describe the evolution of this affection as the best complexity measure was MSE.

#### Entropy of the three stages of a seizure

**Fig. 6:**
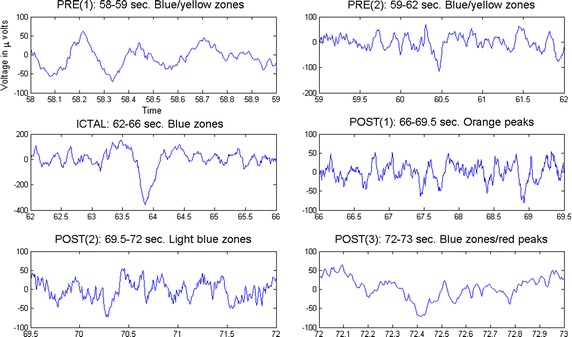
Phases of a seizure separated to study their corresponding complexity curves. Subseparation of stages to compute their MSE curves.

**Fig. 7:**
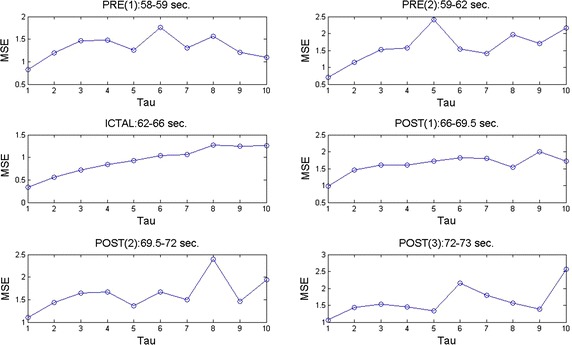
MSE curves for the three stages of a seizure. See text. Complexity curves for each of the sub stages of a seizure.

Consider channel X19 = PZ in EEG2 (2010). A period which contains the three phases of a seizure was analyzed in terms of MSE (Fig. 6). Such interval lasts from second 58 to second 73 in the EEG study. For ease of exposition the preictal duration was separated in two sub phases and posictal stage was split in three sub stages, keeping only one time window for the ictal phenomenon as a result of its brevity. The MSE curves were computed for each of the periods described above and are given in Fig. 7. Notice that during both preictal sub phases the corresponding MSE plots lie above 1 (excluding the initial value at $\tau =1$ where MSE $\approxeq$ 1). There are complexity high peaks for MSE $\ge$ 1.5, indicating so that brain activity is mainly normal. Now, let us analyze the ictal phenomenon. It can be seen that the complexity is low i.e., MSE$<$ 1 for $1 \le \tau \le 6$ and MSE $\approxeq$ 1 for $6 < \tau \le 10$. This indicates that there exists similar patterns during this period of time (Fig. 6) as expected. The first postictal subphase (seconds 66–69.5 in Fig. 6) reflects a recuperation-like MSE curve with values going beyond 1 gradually (Fig. 7). Ulterior intervals of the postictal duration show high complexity again. These set of curves show that MSE is a good option to investigate complicated EEG patterns.

#### Comparison of preictal, ictal and postictal phases in multiple EEG

**Fig. 8:**
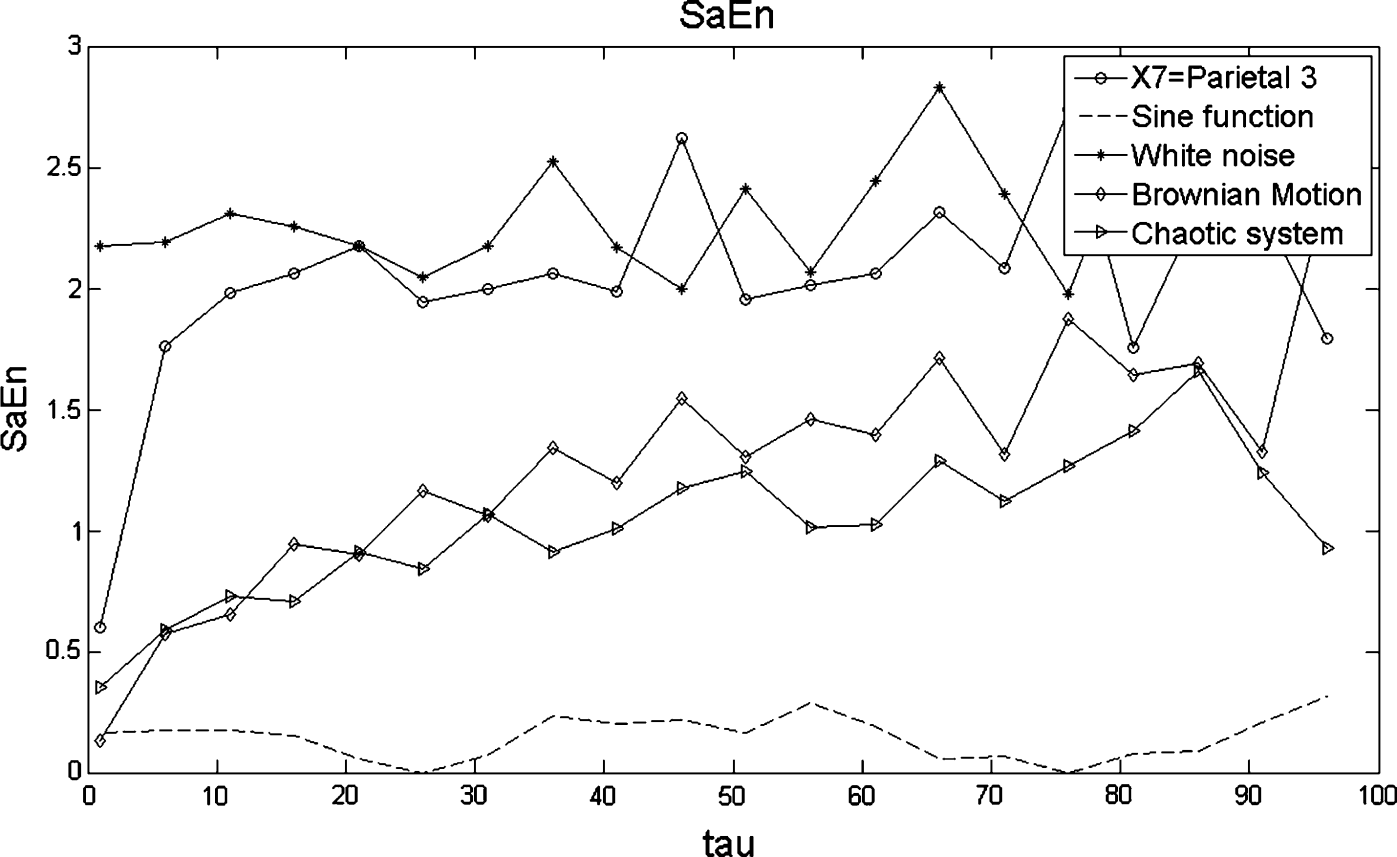
SaEn as a function of $\vec{\tau .}$ SaEn as a function of $\tau$ plotted for five 10,000 samples long signals in order to compare complexity among them.

**Fig. 9:**
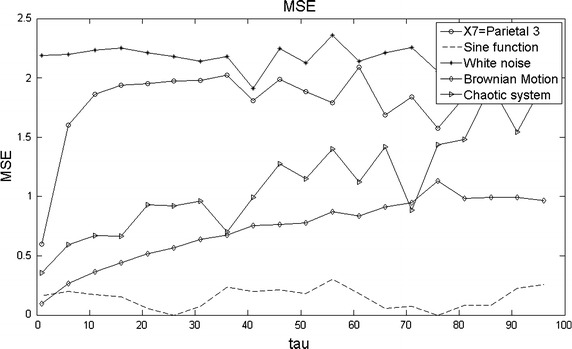
MSE as a function of $\vec{\tau .}$ MSE as a function of $\tau$ plotted for five 10,000 samples long signals in order to compare complexity among them.

Recall that a normal brain activity presents high complexity with respect to simpler signals. This was the case for channel X7 which was compared to a sinusoid wave, a chaotic state variable, Brownian motion and white noise (Figs. 1, 8, 9). In contrast, the preictal, ictal and postictal periods will be analyzed in terms of MSE next for the four years. Since the total number of plots is big, 228, i.e., 19 channels $\times$ 4 years $\times$ 3 seizure phases, the most representative figures were chosen to be those corresponding to the first four EEG channels of all EEG. Each stage was chosen to last approximately the same to make them comparable in time. So, the preictal phase lasts about 13 s, the ictal one, 5 s and the postictal phase, 5 s.

Observe in Fig. 2 how at this preictal stage MSE complexity curves go upwards, indicating a normality tendency (seizure free period and normal distribution of the corresponding set of time series as explained before). Consider now Fig. 3. It is clearly seen that the improving pattern is destroyed during the ictal state. Additionally, the MSE seems to oscillate. This fact means that the brain equilibrium is being altered. Finally, the postictal behavior is given in Fig. 4. There it seems to be a transition from the electrical attack to an improving process. Notice particularly at the end of the horizontal axis an increasing trend in complexity. Again the set of plots coincides with the clinical behavior observed during the EEG recording. We remark that analyzing these plots is much more easier than studying scrolls of printed plots in paper. So, as neurologists coincides, our contribution ended up in a useful support in EEG-analysis.

In Fig. 5 MSE was plotted for four channels. This image can be considered as a portrait of the evolution of the complexity of this DS through 2008–2013 during 1 h and 20 min of EEG recording. Notice how the four plots go up in spite of the affection showing that the little patient achieves a normal mental activity. Observe also how otherwise long rolls of traditional EEG plots are comprised in these entropy curves. It can be conclude as well that our entropy measures are spatially descriptive, i.e., any brain zone can quantitatively be investigated.

#### Multiple long term EEG analysis

**Fig. 10:**
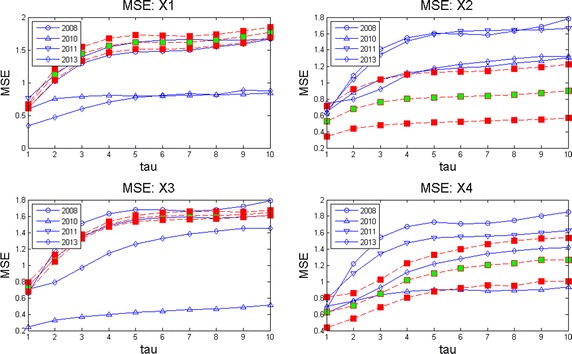
MSE mean, MSE mean plus/minus one standard deviation curves per channel. Multichannel long term MSE plots for all the years considered (X1–X4 shown here). MSE mean, MSE mean plus/minus one standard deviation curves are also shown to help to decide which channel was better in the long term.

Given a set of several EEG studies, it is always difficult for neurologists to decide which one is the best and as a consequence, to explain the evolution of a disease in terms of the whole EEG data set. As we offered at the beginning of this work, our version of MSE is useful to deal with this, displaying multiple long term information in a simpler way than before. Consider for instance the following case. The parents of this kid ask to the neurologist: * which area of the brain is the most affected by seizures through the four years of studies and moreover, how is the evolution of the disease by brain zone through all this time?* These questions can be answered as follows in terms of MSE. Consider Fig. 10 where channels X1 to X4 are given for the four years of studies. In each subplot the mean curve and mean plus/minus one standard deviation curves are also included for each channel. Examining mean and standard deviation MSE plots in the other channels (not shown) it was possible to observe that the most irregular of them i.e., those who exhibit more time-amplitude fluctuations were X2, X4, X7, X10, X14 and X18. On the other hand, by inspecting only the lowest mean complexity it can be deduced that regions X2, X4, X7, X8 and X10 are the worst. Hence, X2, X4, X7 and X10 represent the areas which behave with more irregularities and lowest complexities. Nevertheless, among all of them the channel with lowest entropy and highest variability was channel X2. Hence, X2 = F3 is the region which presents more seizures during these 5 minutes of EEG time. That is why it was decided to show this set of plots for channels X1 to X4.

**Fig. 11:**
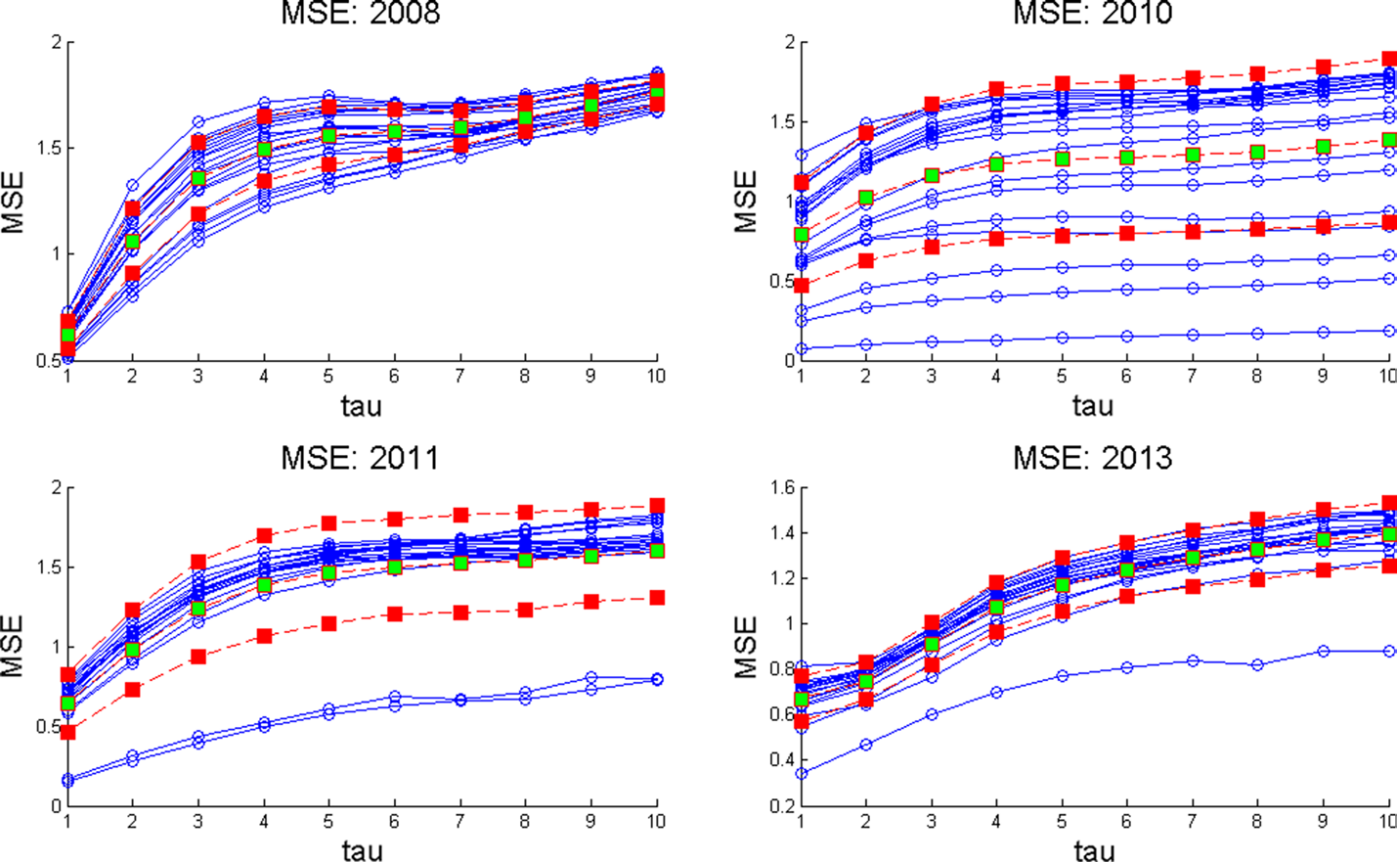
MSE mean, MSE mean plus/minus one standard deviation curves per year. Multichannel long term MSE plots for all the EEG channels per year.

Now consider the parents posing the following question to the child neurologist: * Analyzing the EEG data set, on which year was the kid healthiest and why?*. Naturally, the answer will influence medications, therapies, home-cares, and so on. Scrutinizing Fig. 11 where MSE mean, MSE mean plus one standard deviation and MSE mean minus one standard deviations curves are displayed, it can be observed that the best years were 2008 and 2013 and the worst years, 2010 and 2011. Observing the latter plots it can be deduced that 2010 is the worst year among all. Notice there that the level of the MSE mean is around 1 but the standard deviation curves reflect high fluctuations. Hence, 2010 presents the lowest complexity with the highest variations (predominance of ictal seizures). All of this is valid for the period of time analyzed in all these curves, 5 min of EEG time. Of course, this period of time can be changed. It can be concluded that during 2008, 2010, 2011 and 2013 the worst year of the child was 2010 with X2 = F3 as the most affected zone. These conclusions were in line with clinical observations done by neurologists (Zavala-Yoé et al. 2015).

“3D-MSE: BMSE numerical experiments as clinical interpretation” describes the results obtained during the computation/plotting of BMSE for the experiments explained in “Numerical experiments”. The mathematical-medical link is given in “Mathematical-medical relation: anticonvulsants and BMSE”.

### 3D-MSE: BMSE numerical experiments as clinical interpretation

#### Analysis of 90 s time containing multiple preictal, ictal and postictal phases

**Fig. 12:**
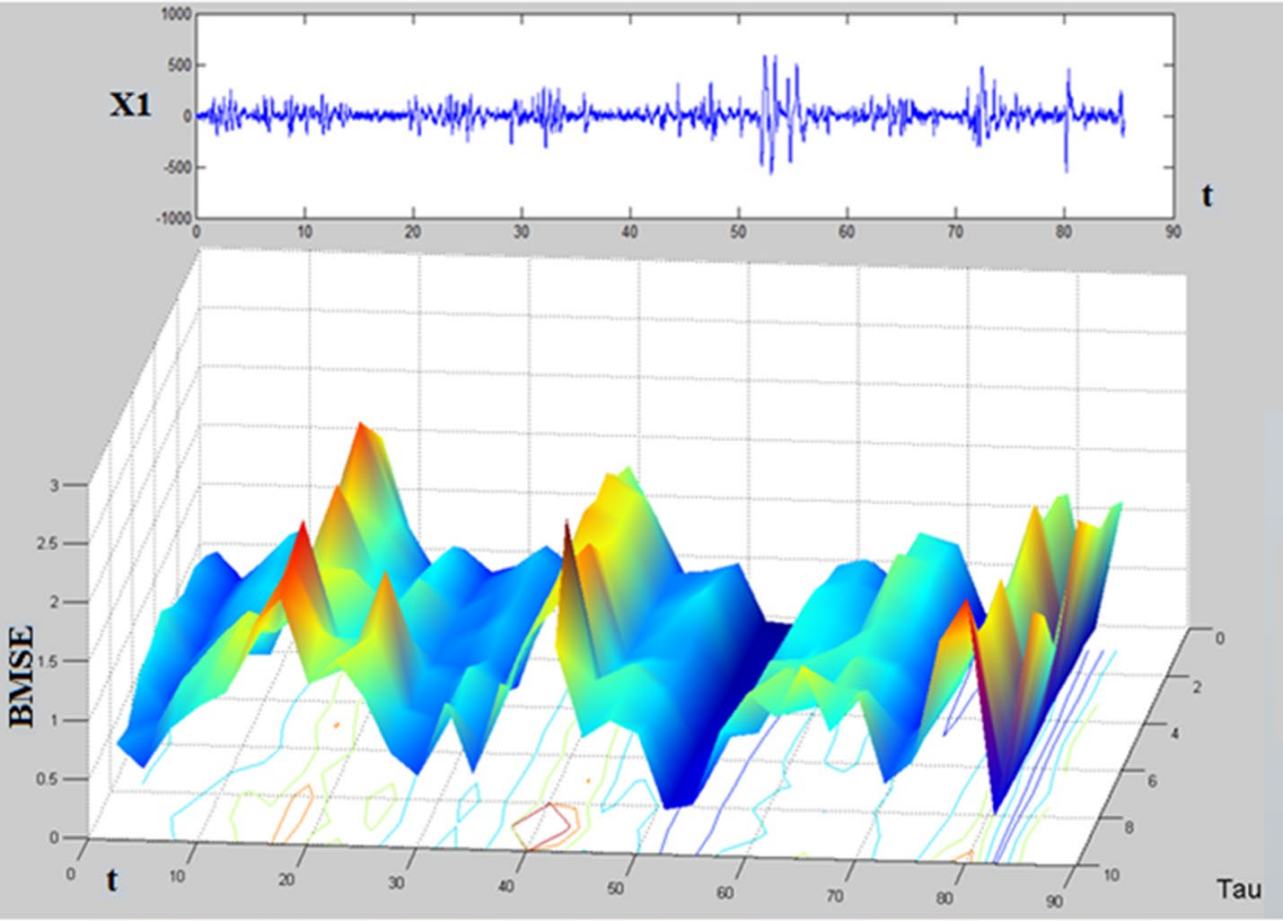
X1 and its corresponding BMSE plot during 90 s time. From EEG2, the first 90 seconds of brain activity are shown in X1 and its corresponding BMSE surface.

In Fig. 12 (upper panel) several preictal, ictal and postictal phases are given from signal X1. In the lower panel, the corresponding SBMSE surface is provided. In general, during a preictal stage, neither neuron synchronization nor high amplitudes are present in the EEG pattern. As a consequence, relative high values of BMSE appear coloring in yellow/red tones the corresponding region. Later, during the ictal phase, the neurons are synchronized and the voltage amplitude increases a lot, hence blue tones color the SBMSE plot. Finally, throughout the postictal part, amplitudes come down and blue tones tend to disappear in the SBMSE surface. The following details can be appreciated in the aforementioned Fig. 12. It can be seen that there are periods where the pikes and high frequency activity are greater than in other sections of this signal. Analyzing for instance the first fifteen seconds of X1, it can be seen that some high voltage peaks are present. Comparing this period of time with the BMSE plot below, it can be appreciated that the complexity goes up until second 15, where $BMSE\approx 2$. It is also interesting to observe that at second 15, and going towards $\tau$ axis (going ”inside” the page), there are peaks colored in red/yellow, meaning that the complexity is really high at second 15 (notice the BMSE scale). This fact matches with the information provided by the EEG in channel X1 shown above. Later, from second 15 to second 20, although the complexity still is relatively high, it starts to go downwards (observe also this towards $\tau$). Approximately at second 28, complexity falls down to its lowest value in this period. This coincides with the peak pointing downwards in the X1 plot. Notice also the blue color in the BMSE at second 28 ($\tau$ direction) which says that for all values of $\tau$ the complexity is low. From second 30, the BMSE tries to improve (notice the yellow peaks in the 3D surface), reaching its highest value approximately at second 40. High frequency and high voltage peaks appear after second 40 and until second 55 (see X1 graph in the current image). This events are displayed in the BMSE surface where BMSE ≤ 1 for the period from second 45 to second 55, approximately. Next, up to the second 70, the X1 amplitude remains normal, fact reflected by the BMSE surface. The highest value of BMSE is reached at second 75, because a little bit later some irregular activity shows up until second 82, approximately. Finally, after second 82 the brain activity looks well in channel X1 with BMSE going upwards and with yellow/red tones in the $\tau$ direction.

#### Comparison of BMSE and MSE in X19 of EEG2 (2010)

**Fig. 13:**
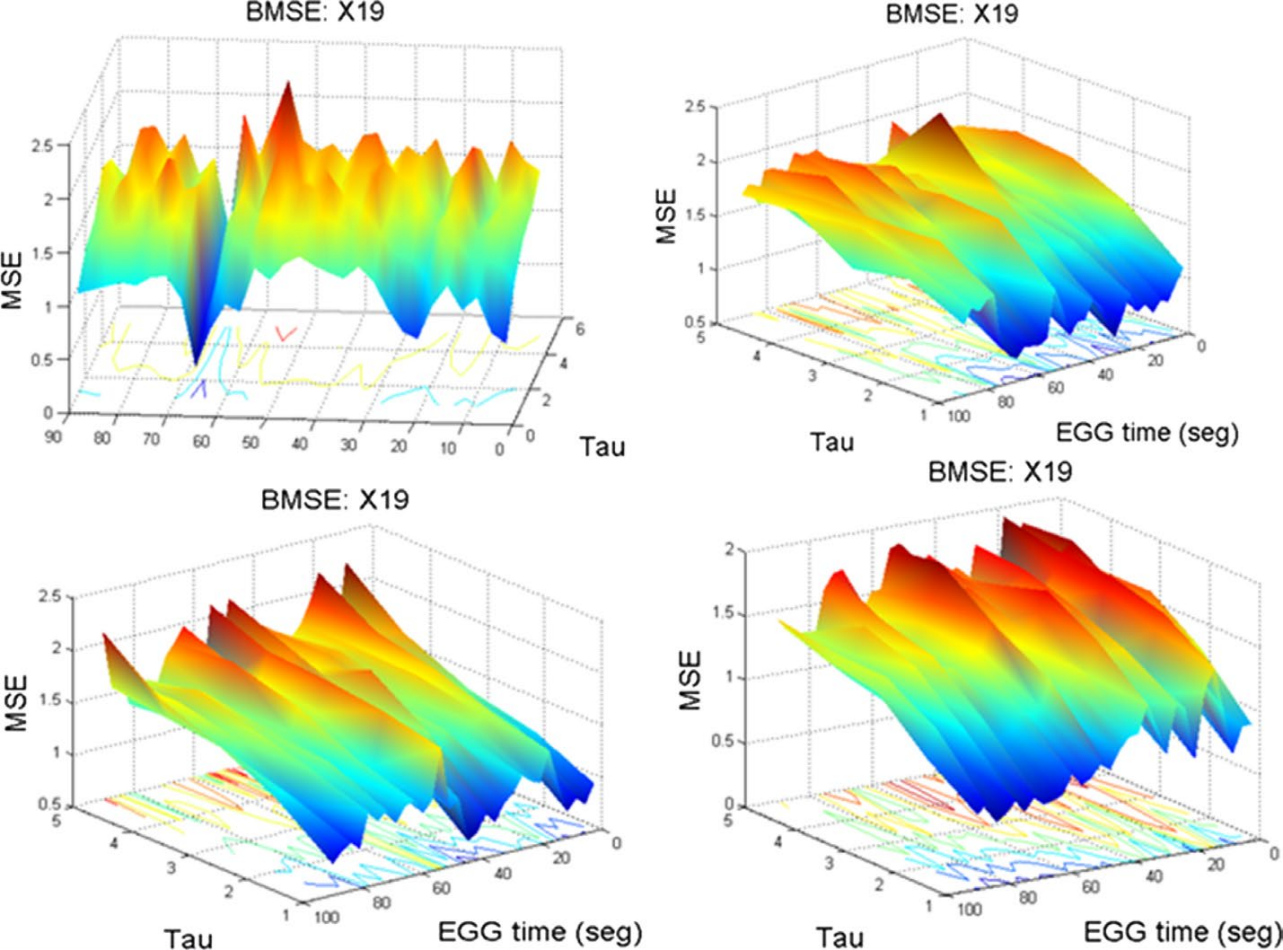
Bivariate $\vec{MSE(t,\tau )}$ complexity corresponding to 6 min time in X19, EEG2 (2010). Compare this subfigure (1,1) with Fig. 6, panel (2,1); a 2D and a 3D viewpoints of the negative peak Here, each surface comprises 90 seconds of EEG2.

Signal X19, EEG2 (2010) was already described by MSE in “Entropy of the three stages of a seizure”. Now, that signal will be studied by BMSE (SBMSE in four phases, 90 s each). Recall that there is an important negative peak during the ictal phase (Figs. 6, 7) which is now shown by SBMSE surfaces (see Fig. 13). Subpanel (1,1) of the latter image says that the lowest complexity of the surface appears at second 65 approximately. Blue zones means low complexity (BMSE  $\le$  1). Yellow/red tones means high complexity, predominant in the remainder surfaces. Notice that the lowest parts of the surfaces which appear in subfigures (1,2), (2,1) and (2,2) exhibits blue areas. This means that the complexity is low for the original time series and small values of $\tau$. However, when $\tau$ becomes bigger, the complexity (BMSE values) tend to be high (bigger than 1) and ripples appear. In spite of the aggressive seizure appeared at second 65, the background brain activity tends to be of high complexity, i.e., the brain recovers and tries to go on. This fact coincides with the results given by MSE.

#### Description of long term BMSE complexity in 2.5 h time of channel X1 in EEG4

**Fig. 14:**
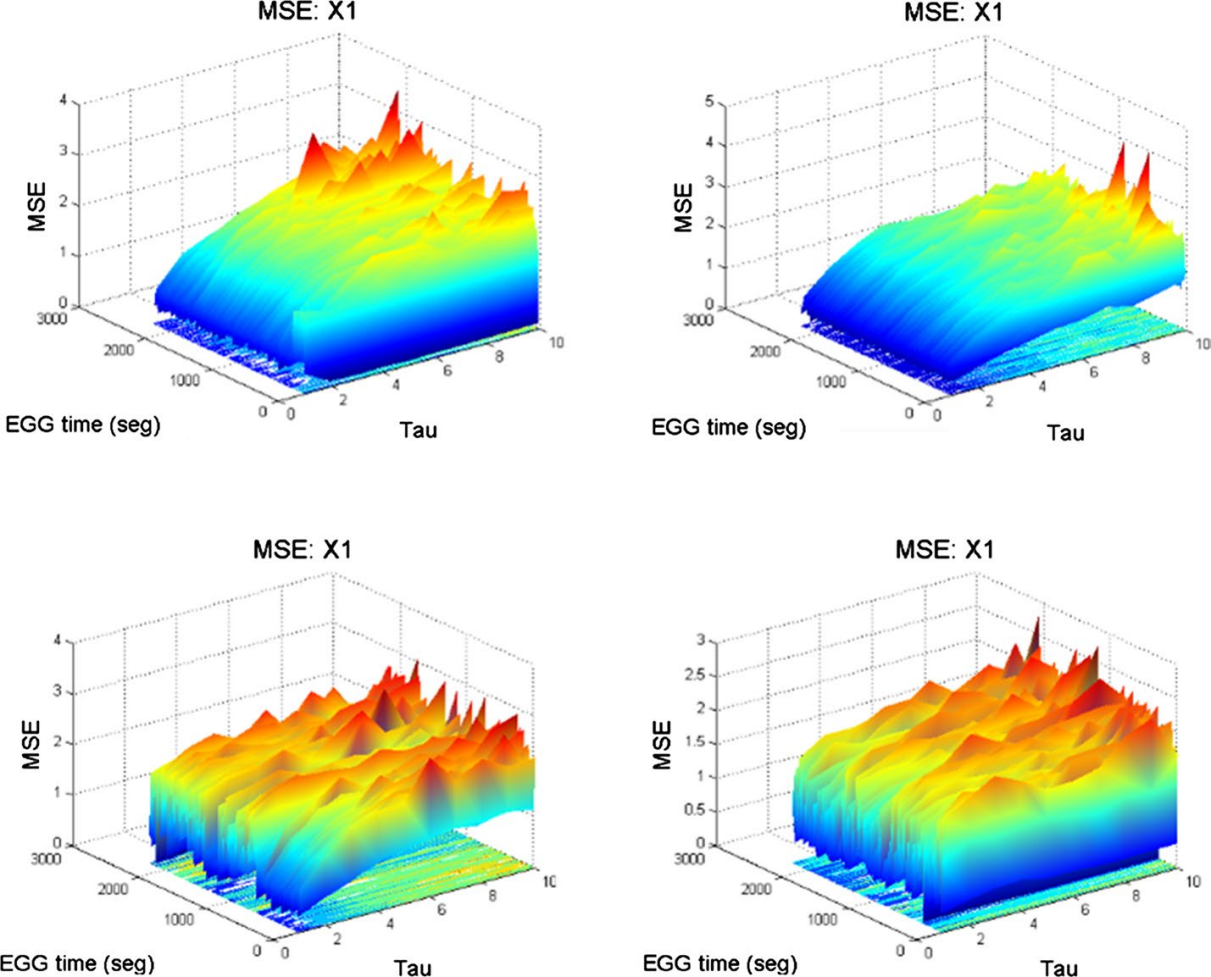
Bivariate $\vec{MSE(t,\tau )}$ complexity corresponding to 2.5 h of an EEG study in channel X1. Each figure comprises 37.5 min of an EEG record. Long rolls of traditional EEG plots may be supported by this kind of graphs.

In the last experiments the BMSE computation has been done for relative short times, a matter of minutes. BMSE is also useful to study very long term patterns of complexity of EEG signals. Consider for instance Fig. 14 where 2.5 h are split in four 37.5 min long 3D graphs. The left upper panel shows the first 37.5 min of complexity evolution. It can be seen there that the blue regions of low complexity are relatively scanty and appear at the bottom of the surface. Note the light blue line which appear at BMSE = 1. Upper regions are colored in yellow/green indicating higher values of BMSE. At the end of the figure red spikes can be observed. All of this says that the first 37.5 min are relatively good for the kid. The figure corresponding to the following 37.5 min (70 min up to now) presents bigger blue zones than the first one in the right upper panel. Moreover, the surface looks rather flat, yellow/green areas are not big and there are some red spikes. There are not many seizures but the complexity (in general) tends to be low in this period of time. The left lower panel shows a recovery of the above mentioned low complexity because yellow/red areas can be easily observed. Nevertheless the surface looks curly as a result of a constant irregular activity. The latter means that although the child suffers a constant electric state (see the ripples) this kid performs high level tasks for a human being (she plays,she watches TV, etc.). Finally for this image, in the right lower figure, the latter behavior persists, indicating that the child lives (almost) a normal life. He might not exhibit seizures but he really suffer them. This is confirmed by both lower panels of this figure. It is very remarkable that neurologists have coincided with this sad characteristic of this kid.

#### Comparison among X17, X18, X19 and X20 during 10 minutes of EEG4 time

**Fig. 15:**
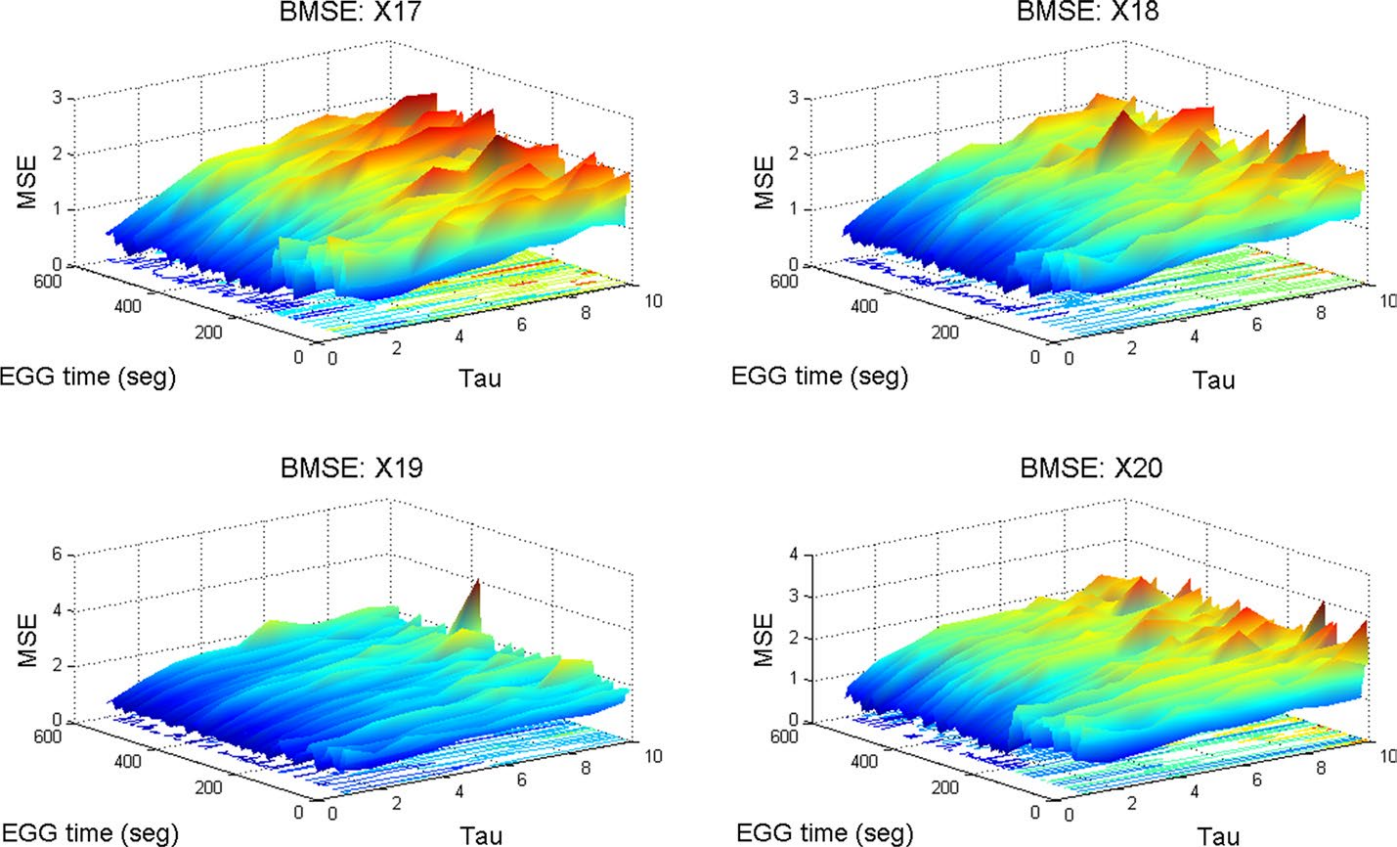
BMSE in channels X17-X20 in EEG4. This image reveals that channel X19 is the worst one during the 10 min of the study in EEG4. Note low BMSE zones (approximately equal to one) which indicates low complexity for this period of time.

In this case, four different channels were compared in order to observe their long term BMSE complexity during 10 min of EEG time. It is possible to notice that X19 is the worst one because its surface remains almost flat (slightly above 1 in the BMSE scale) indicated by vast blue regions. See Fig. 15. These surfaces make comparable patterns that otherwise would not match as a result of the length of the plots and the quantity of EEG involved. If we reflect that an epoch (an EEG page) lasts typically 10 seconds, 10 minutes of an EEG study means that we have 60 pages of voltage-time graphs. Such information is condensed in MSE and BMSE plots.

#### DBMSE: computational animation of 2.5 h of BMSE evolution in EEG4 

The multiple figure described above in “Description of long term BMSE complexity in 2.5 hours time of channel X1 in EEG4” was animated by a MATLAB program (see Algorithm 3) which permits to observe and follow the behavior just described. In this case, 2.5 h of an EEG recording can be comprised in four frames movie which shows gradually how DS evolves. Naturally, the animation can be as finer or broader as the user wishes. The subjective interpretation of an EEG (see “Introduction”) can be supported by BMSE plots as it has been explained in this section. It is possible to identify low complexity zones, which correspond in the EEG graph to repetitive patterns with low amplitudes. Red/orange regions in BMSE surfaces means high complexity patterns in the EEG record as explained before. Naturally, a DBMSE can also be obtained for the set of figures (or more if it is desired) given in “Comparison of BMSE and MSE in X19 of EEG2 (2010)”.

### Mathematical-medical relation: anticonvulsants and BMSE

The results just showed in “3D-MSE: BMSE numerical experiments as clinical interpretation” were considered to be the most representative ones for this case of DS as a result of the neurologists observations, particularly the frontal fuzzy focus explained at the end of “EEG databases”. The 3D plots of BMSE as well as the computational animations were done for all the EEG channels in the database. Although these results were not included for space restrictions, all of them coincide with the observations done by neurologists. It was confirmed that 2008 and 2013 were worse years than 2010 and 2013. This could be deduced from all the BMSE surfaces and animations which showed less ripples and blue zones in 2008 and 2013 than in 2010 and 2011. Anticonvulsants therapeutic action is manifest in yellow/red zones and peaks of high complexity in static BMSE and dynamic BMSE (see Table 1 and “Anticonvulsants information”). In spite of a constant discharge state in the kid, the successful combo ethosuximide, levetiracetam, clonazepam, lacosamide and lamotrigina resulted to be good until 2013. The red/orange/yellow zones confirm this fact with high complexity activities in the kid as playing computer games, going to school, etc. Nevertheless there does exist an impairment between the kid’s development and the rest of the children of the same age, but psychologists and neurologists agree that the child is improving, slowly, but improving at least until 2013. Long term static and dynamic BMSE also coincide with this as well as our computations of MSE.

## Conclusions

Four entropy parameters were investigated: ApEn, SaEn, a modified version of MSE and CMSE. These nonlinear measures confirm low complexity not only during ictal stages but also throughout long periods in some brain regions. Again, this comes as a result of a constant discharging state. Among the aforementioned statistics, our modified MSE was chosen as the best one to deal with this type of children epilepsy. In this sense, it was found out that massive information contained in traditional long rolls of EEG can be condensed in very simple MSE plots. Moreover, the latter is also possible considering not only one EEG but also many. So, the information contained in multiple long term EEG can be resumed and made suitable for self comparisons through time in terms of MSE graphs. We have to reflect that such massive information may induce subjective interpretation by neurologists. Hence, MSE can quantify objectively from which brain area came the more aggressive discharges as well as on which year. These facts have not been answered specifically and quickly by medical experts under the conditions explained. Besides of these advantages, the evolution of one patient can be compared objectively with respect to others.

After applying MSE to the set of 80 channels it could be determined that 2010 was the worst year for the kid with X2 = F3 as the most discharging region. Later on, in this work we will develop and asses another entropy statistic which will be applied to this type of CCRE.

Finally, DS was investigated in terms of static and dynamic BMSE as complexity measures. This fact offers a significant advantage in long term interpretation of this disease by studying complexity measures over traditional observation of long rolls of EEG plots. Long term and very long term evolution can be studied with the proposed BMSE. The red/orange zones in static and dynamic BMSE indicated that the kid is progressing in spite of this difficult struggle (Zavala-Yoé et al. 2015).

## Future work

As a future work, more databases recorded from other children have to be included in order to determine whether it is possible to use BMSE as a DS marker. It will be investigated if BMSE has a finger print in DS (and also in other CCRE). Moreover, the use of complexities and specially BMSE can be extended to other types of CCRE. This work is presently being developed. On the other hand, the possibility of predicting seizures by means of MSE or BMSE has to be explored. High frequency components complicate prediction of EEG time series, but MSE and BMSE do not show this problem. However, it is necessary to have some thousand samples to compute an Identification Model, but MSE and BMSE are not that long. Hence, a trade off has to be found. Actually, in parallel with the results shown in this work, prediction simulations were obtained using NLARX (Non Linear Auto Regressive with eXogenous inputs) models working directly with the EEG time series but since this DS is multifocal, 20 times series have to be predicted at the same time complicating the scenario (Zhang and Ljung 2007). NLARX is a good prediction model but for one or two steps ahead and only one time series. As more steps ahead and channels required, as less precise are the prediction outputs (Zhang and Ljung 2007). Results were also obtained predicting MSE and BMSE with NLARX models but the outputs were not satisfactory. These algorithms are currently being extended to improved versions.

Researching deeper this topic we hope to support better to neurologists to clarify multiple long term EEG in CCRE. May be we can contribute to find a definitive clue that can determine the origin (and maybe the cure) of this cruel pathology which silently affects to many children.

## Methods

### Design of the study

A very rare case of DS known by its medical complexity (CCRE) is considered here in order to be investigated mathematically. DS is described by a set of four EEG (80 time series) which were be investigated by four complexity measures, implemented in our three-dimensional arrays (ApEn, SaEn, our refined MSE and CMSE). Among the latter, our improved version of MSE ended up to be the best to describe the evolution of this DS. So, this non-linear statistic was used to answer the questions posed in “Contributions as goals” successfully. First, MSE was used to make multiple long term EEG information *comparable* during the three phases of a seizure. Second, 2D-complexity plots (MSE plots) were able enough to explain quantitatively which region of the brain was the most affected and which year was the healthiest during the time of the study considered. Proceeding as usual in EEG analysis by neurologists, these conclusions would not have been possible to obtain. Third, since it was determined that the most convenient entropy to analyze EEG was MSE, it is proposed here to construct another complexity measure referred to as Bivariate Multiscale Entropy (BMSE) which will be a function of time and scale factor time $\tau$, i.e., a 3D-measure. The evolution of a channel with respect to time will be displayed as *MSE*(*t*). $MSE(\tau )$ will show how the EEG signal varies its regularity pattern conforms vector $\tau$ varies. So, BMSE will be defined as $BMSE=MSE(t,\tau )$. Since BMSE is a function of two parameters, the corresponding BMSE will be a 3D plot (SBMSE). Moreover, it will be shown here that putting several BMSE plots together at the same reference frame, it will be possible to produce a useful computational animation (DBMSE) which will give long term information about EEG complexity. Note 2 about nomenclature. Since $BMSE=MSE(t,\tau )$ these terms are used as synonyms.

### Subject,EEG databases and record of anticonvulsants

#### Subject

A female child whose age ranges 8–12 years old was investigated. It is noteworthy mention that only one patient was considered here because DS is a relatively uncommon affection (Stephani 2006; Doege et al. 2014; Kelley 2010). A yearly average of 2.4 children with DS are admitted to this hospital in Mexico City. As far as we are concerned, this is a unique case at this hospital with such severe symptomatology. As a consequence, the child has not been cured yet. This is the reason of considering only one child in this investigation.

#### EEG databases

**Fig. 16:**
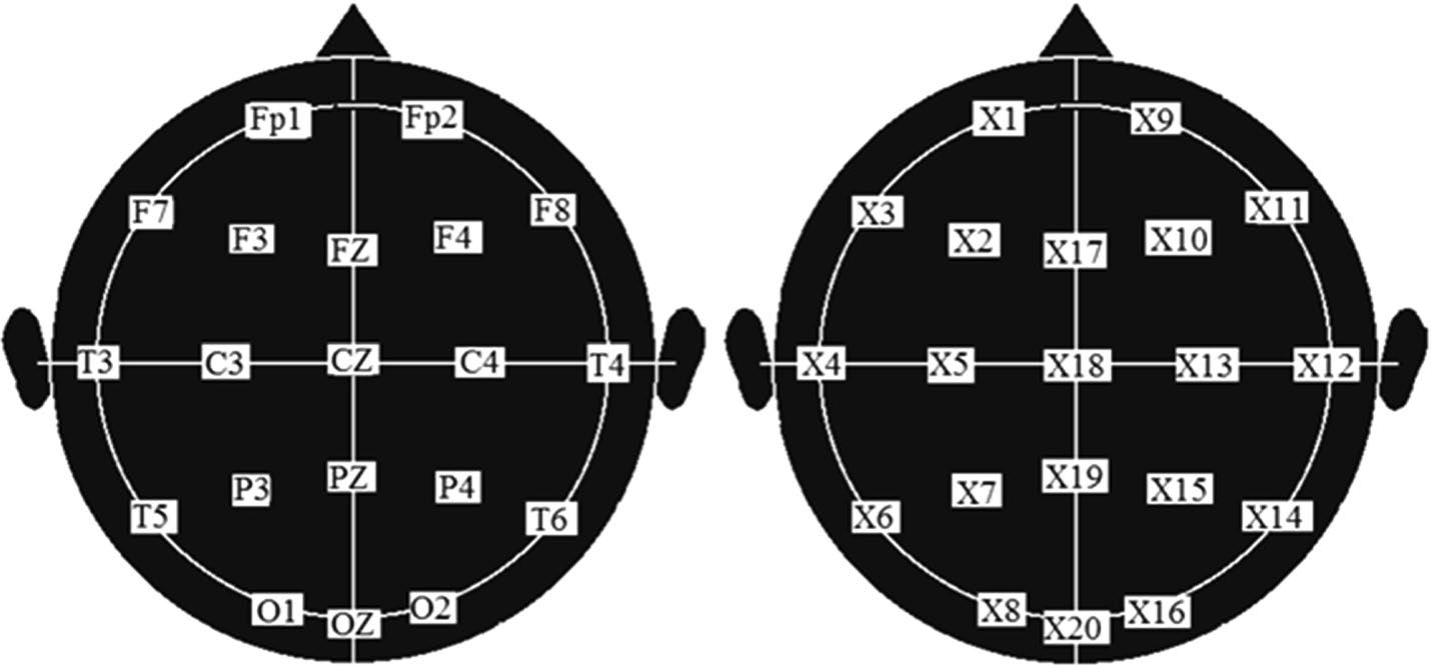
International std. 10–20 system for electrode positions (head seen from *top*). Each position is called channel and corresponds to one time series.

Although the disease history of the child starts in 2006, the database consider only 2008, 2010, 2011 and 2013. Years before 2008, i.e., 2006 and 2007 were not available and missing years from 2008 to 2013 were not accessible for administrative purposes. Hence, four EEG compose the present database: 2008, 2010, 2011 and 2013 so they are referred to as EEG1, EEG2, EEG3 and EEG4, respectively. Each of them is conformed by 20 channels. The entire set of the four EEG clearly shows typical features of DS although some clinical manifestations are not consistent with a typical DS (overlapping). The EEG were recorded according to the international 10–20 system with 7 mm, 7 $\mu$v calibration (Gil-Nagel 2001). As known, this system identifies electrodes with the following identifiers: The letters F, T, C, P and O stand for frontal, temporal, central, parietal, and occipital lobes, respectively. Although there is no central lobe letter ”C” is used only for identification purposes. Letter ”z” means zero and it refers to an electrode placed on the midline. The right hemisphere electrodes are identified with even numbers (2,4,6,8) whereas odd numbers (1,3,5,7) refer to those on the left hemisphere. Fp means frontal polar electrode. That is why our electrode positions were: Fp1, F3, F7, T3, C3, T5, P3, O1, Fp2, F4, F8, T4, C4, T6, P4, O2, FZ, CZ, PZ,Oz (twenty) (Gil-Nagel 2001; Shorvon 2011), see Fig. 16. Although BMSE did not show memory restrictions as it was reported in “CMSE analysis”, the number of channels considered in the numerical experiments were 19 as it was for the flat entropies (ApEn, SaEn, MSE, CMSE). So, for sake of clarity in graphics, these channels were renamed as $X_{i}$, i = 1,…,19. The sample frequency was 200 Hz corresponding to 5 ms of sample time. A/D converter resolution was 16 bits.

The EEG records are matrices of different sizes, thousands of rows (samples) by 19 columns (channels). There were no abnormal body activity nor evident seizures exhibited during recording of EEG1 and EEG3. However, this was not the case during recording of EEG2 and EEG4. See “Anticonvulsants information” and “Mathematical-medical relation: anticonvulsants and BMSE” where the latter is confirmed in this second part.

Neurologists reported that it would have been great to find the focus of this disease because the discharges spread throughout the brain very quickly. Sometimes a surgery can extirpate the focus area in order to finish the affecting discharges. But in this child, neurologists found that this epilepsy is multifocal and a surgery is not reliable. In spite of being a multifocal problem, one of the main origins of seizures is the frontal lobe. Nevertheless, this focus is fuzzy in the sense that there are other small foci in this region.

#### Anticonvulsants information

Table 1 provides information about the main anticonvulsants prescribed for the kid during 2008–2013. In that table the abbreviations used are explained in the List of Abbreviations, at the end of this document. Details about those antiseizures can be reviewed in (Doege et al. 2013; Stephani 2006; Steinhoff and Bast 2013; WHO 2013a, b).

**Table 1:** Anticonvulsants per year

Anticonvulsant	Year	Comments	Seizures	MSE	BMSE
IMI, PA	2006	First medicines consumed. Stopped suddenly	NA	NA	NA
VPA+, TPM, CLB, VPA	2007	Beginning of VA	NA	NA	NA
VPA, LTG, TPM, VPA+, CLB, LEV, ATX, ESM, PSE, MDZ	2008*	Pancreatitis. Beginning of ESM,LEV. ATX worsen seizures	$\surd$	OK	OK
TPM, LEV, LTG, PSE, ESM, CZP, ZNS	2009	ZNS useless. Retirement of ETS worsen seizures	NA	NA	NA
ZNS, ESM, CZP, LEV, CZP	2010*	CZP useless. Seizures worse	$\times$	Bad	Bad
CZP, LEV, LTG, ESM, PB	2011*	PB useless	$\times$	Bad	Bad
LCM, ESM, LEV, LTG, CZP	2012	LCM shows up.ETS retired again worsening seizures	NA	NA	NA
LCM, LEV, LTG, PRM, CZP, Q10, ESM	2013a*	Gastritis aggravate	$\surd$	OK	OK
LEV, ESM, LCM, LTG, CZP	2013b*	Idem	$\surd$	OK	OK

$\surd$ and $\times$, a relative good and poor control of seizures, respectively; NA, not applicable.

* an EEG was recorded in that year and is used in this work

It was very difficult to express the whole and detailed information available about dates, dosages, duration of medicines treatments, side effects and so on in only one resumed table. However, so far the information displayed in Table 1 is good enough for the purposes of this work. It is also remarked that although the medicines cited above are produced by international companies, some of them have local versions in Mexico City. Examples are LTG and ESM. From the table, it can be seen that ESM is useful for the kid but the side effect is gastritis (WHO 2013a, b; Steinhoff and Bast 2013). Stopping consumption of this product exacerbates seizures as reported above. Q10 was prescribed as an emergency help but it was useless. It is also remarkable that phenytoin is forbidden for this child because it causes her status epilepticus (SE) (Doege et al. 2014). The last two columns in this table indicate the univariate MSE and BMSE for each year of the EEG. Notice how this parameters coincide with all the other information provided by this table. Since MSE and BMSE are curves and surfaces, respectively, the value ‘ok’ means that the complexity was mainly high (see “Results and discussion”).

#### Evolution of DS according to medical treatment

The following description is complemented in “Mathematical-medical relation: anticonvulsants and BMSE”, where the mathematical connection is established. Consider Table 1 Seizures history starts at the beginning of 2006 with just a few Absence episodes which seemed to be normal. Later, at the end of 2006, some irregular EEG activity was detected. The diagnosis was brain immaturity (Michels et al. 2011) which was supposed to be not serious. However, presumably, sudden retirement of IMI and PA exacerbated seizures. Since the affection was evolving, 2008 was worse with respect to 2006 and 2007. In 2007 VPA+ was used to mitigate myoclonic and atonic seizures which had showed up. Since VPA+ did not reduced the seizures, VPA was prescribed in 2008 and was used during that year until pancreatitis (pancreas inflammation) complicated the treatment. Pancreatitis is a very dangerous disease which may affect people that are medicated with VPA+ or VPA (Hartford Hospital Evidence-based PracticeCenter 2011; Steinhoff and Bast 2013; WHO 2013a, b). In this case, people have to be sent to a hospital for medical attention. After two weeks of admission, during which the child received midazolam, seizures vanished. In that time, the child suffered a bit of what was diagnosed as attention deficit and hyperactivity syndrome (ADHD) and atomoxetine was prescribed to help (WHO 2013a, b; Steinhoff and Bast 2013). Nevertheless, this drug worsened seizures again and it had to be retired. From the comments above, in 2008 seizures were partially controlled and the kid had a relative good control of her epilepsy in spite of the pancreatitis and the side effects caused by atomoxetine. 2009 is a missing year in the EEG database of this work and could not be studied with BMSE. However, during some months of that year zonisamide was tried with no success. In addition, ethosuximide was retired exacerbating the seizures. This fact continued in 2010 (available EEG) when clorazepate dissodium came into picture with no control of seizures. Summing up, 2010 was a bad year for the kid as a result of a poor control of her epilepsy. Next, in 2011 a special combination of anticonvulsants was conceived. As a result, a better control of the seizures was achieved with respect to 2010. In 2012 ethosuximide was gradually stopped with the consequent worsening of seizures. So, although 2012 is missing in our database it certainly influenced the future and was not a good year in terms of epilepsy control. Finally, in 2013 a right amalgam of medicines was found after a very long and painful trial and error process. It is concluded that 2013 was a relatively good year. It is interesting to notice that this successful combo was already used since 2011 (a relative good year) but mixed with other medicines. Summing up, 2008 and 2013 were good years and 2010 and 2011 were not. This observations coincide with the conclusions thrown by MSE in “MSE analysis”.

### Description of comparisons: complexity of multiple long term EEG

The evolution of epilepsies as DS is explained traditionally in terms of the multiple long term EEG. However, the amount of information contained in such set of studies is unhandy and may become subjective. That is why it is proposed in this work to assess a set of complexity measures in order to choose the most suitable to describe multiple long term EEG. The set of entropies were assessed in a set of test signals described in “Test signals”. One of them is an EEG-signal which qualifies as normal for the work period chosen.

One way to define complexity of a dynamical system is by means of its *entropy*. In this context, entropy is the rate of information production (Richman and Moorman 2000). Pincus (1991) developed the theory for a measure of regularity, the rate of generation of new information that can be applied to clinical data. Pincus named this measure *approximate entropy, ApEn*, having as a goal to measure system complexity. From ApEn, some other entropies have been proposed (Chon et al. 2009; Richman and Moorman 2000; Costa et al. 2002; Shuen-De 2013), and our versions of MSE and CMSE used in this work (“ApEn, SaEn–CMSE analysis”) as well as our BMSE (SBMSE and “Description of comparisons: static and dynamic BMSE”). We remark that we analyzed all EEG-channels of our entire database with all the statistics (complexity measures) described here. However, from our 320 graphs (80 times series $\times$ 4 entropy measures) we only show relevant figures.

#### Test signals

In total, a set of five test signals were used to compare complexity among them:

1. Sine function, f = 1 Hz. The least complex signal.
2. Brownian Motion. A self similar signal but more complex than a sinusoid (Mikosch 1998).
3. Chaotic system: One state variable of a Roessler system (Strogatz 1994).
4. EEG channel, X7, parietal 3 (a brain signal which behaves normal).
5. White noise.

Every test signal is 10,000 samples long, equivalent to 50 s time. We want to show how normal brain activity looks like in terms of complexity (the four entropies already mentioned) with respect to other typical signals. As expected, as simpler the signal as lower its complexity measures.

#### Complexity as univariate multiscale entropy, MSE

A time series is considered more complex than another if for majority of the vector scales $\tau$ its entropy values are higher than other. In addition, a monotonic decrease of the entropy values with respect to scale factors reveals that the signal only contains information in the smallest scale.

It is known that one way to define complexity of a dynamical system is by means of its *entropy*. In this context, entropy is the rate of information production (Richman and Moorman 2000). Pincus (1991) developed the theory for a measure of regularity, the rate of generation of new information that can be applied to clinical data. Recall that ApEn was improved in a new algorithm developed by Richman and Moorman (2000) where the new statistic was named sample entropy (SaEn). Later, such SaEn was refined by Costa et al. (2002) and the resulting parameter was called multiscale entropy (MSE). This MSE was improved here (see “Multiscale entropy, MSE”) and later (“Description of comparisons: static and dynamic BMSE”) an enhanced version of it is referred to as BMSE. The complete EEG data base was analyzed with this new BMSE algorithm and the conclusions were agree with traditional EEG time-amplitude graphs. So, the MSE complexity plots are included below in order to be compared with their corresponding BMSE plots. In all the algorithms, given a vector $V_{1}$, semicolon means to construct a subvector $V_{2}$ which starts in entry $V_{1}(a)$ and finishes in entry $V_{1}(b)$. If an increment $\Delta V$ is specified, $V_{2}$ is written as $V_{2}=[V_{1}(a): \Delta V: V_{1}(b)], a<b$. If $\Delta V$ is not specified, then it is assumed to be one. Our algorithms are based in 3D-arrays storing (Zavala-Yoé 2008).

#### Aproximate entropy, ApEn

Pincus designed an algorithm to approximate a computation of the so called Kolmogorov-Smirnov (KS) entropy (see Pincus 1991 and references therein). The KS-entropy formula is rather abstract and involves numerical disadvantages:

1 $$\begin{aligned} KS_{Entropy}=\lim _{r \rightarrow 0} \lim _{m \rightarrow \infty } \lim _{N \rightarrow \infty }\left[\Phi ^{m}(r)-\Phi ^{m+1}(r) \right]\approx ApEn \end{aligned}$$

The meaning of the variables in Eq. 1 will be clear next. ApEn is widely used to understand complexity of physiological data (Pincus 1991; Chon et al. 2009. With regard to compute ApEn, given a monovariate time series *X* of length N, the basic algorithm (Pincus 1991) firstly creates N-m+1 sub series *x* of length m from *X*. Secondly, computes a distance between two consecutive vectors as the maximum difference in their respective scalar components (see Pincus 1991 and our algorithm below). Thirdly, a parameter $C_{i}^{m}(r)$ measures within a tolerance *r* the regularity or frequency of patterns similar to a given pattern. Finally, parameters $\Phi (r)^{m}$ and $\Phi (r)^{m+1}$ are calculated to represent the average stability of these similar patterns on incrementing. ApEn is computed as $\Phi (r)^{m}-\Phi (r)^{m+1}$. See details in (Pincus (1991); Chon et al. 2009); Richman and Moorman 2000). So, ApEn measures the logarithmic likelihood that runs of patterns that are close remain close on next incremental comparisons within a tolerance r. This idea is referred to as *complexity* Pincus (1991). Thus, summing up, an ApEn complexity plot is obtained in two basic steps. First, by downsampling a time series at a scale factor vector $\tau$, which will produce *l* sub time series (see step 3 in Algorithm 1 below). Second, an ApEn is computed for each sub time series. Finally, the complexity graph is obtained by plotting $\tau$ vs. ApEn (see Algorithm 1). A time series is considered more complex than another if for majority of the vector scales $\tau$ its ApEn values are higher than other. In addition, a monotonic decrease of the ApEn values with respect to scale factors reveals that the signal only contains information in the smallest scale. This is confirmed by the fact that its standard deviation $\sigma (ApEn)$is inversely proportional to the length N of the time series (Richman and Moorman 2000; Pincus 1991).

Think for instance in the following example. Assume that we want to analyze the complexity of an N-samples long signal given by $$X,=...,11.74,1.25,-4.55,$$$$11.74,1.25,-4.55,11.74,1.25,-4.55,...$$, $$N=51$$. Assume that $$m=2, r=3$$. In this case the sequence of sub vectors $$x(i),i=1,...,N-m+1$$ of length *m* (see Algorithm 1) is given by $$x(1)=[11.74 \quad 1.25]$$, $$x(2)=[1.25 \quad -4.55 ]$$, $$x(3)=[-4.55 \quad 11.74]$$, $$x(4)=[11.74 \quad 1.25]$$. Now, the distances are evaluated in such a way those vectors which satisfy the constraint $$d=max{{|x(i),x(j)|}} \le r=3$$ will be counted. Observe that $$d(x(1),x(2))=max{|1.25-(-4.55)|,|11.74-1.25|}$$ = $$|11.74-1.25|\,=\,10.49>3$$ (not counted). Similarly, $$d(x(1),x(3))=16.29>3$$ (not counted either) and $$d(x(1),x(4))\,=\,0<3$$ (counted). Proceeding this way, we realize that vectors which satisfy $$d(x(1),x(j))<3$$ are *x*(1), *x*(4), *x*(7), ..., *x*(49) (seventeen elements). Next, $$\Phi (m)$$ will be constructed as

2 $$\begin{aligned} \Phi ^{m}(r)=\frac{1}{N-m+1}\sum _{i=1}^{N-m+1}\ln (C_{i}^m(r)) \end{aligned}$$

The first term of Eq. 2 is $C^{2}_{1}(3)=17/50$. Continuing this way $\Phi ^{2}(3)$ will be

3 $$\begin{aligned} \Phi ^{2}(3)=\frac{1}{50}\sum _{i=1}^{50}\ln (C_{i}^2(3))=0.333666 \end{aligned}$$

Analogously for $\Phi ^{m+1}$ we obtain

4 $$\begin{aligned} \Phi ^{3}(3)=\frac{1}{49}\sum _{i=1}^{49}\ln (C_{i}^3(3))=0.333666 \end{aligned}$$

Finally, $ApEn=\Phi ^{2}(3)-\Phi ^{3}(3)=0.000033 \approx 0$. Hence, this periodic signal (or at least, the part considered here) is not complex. The complete and detailed algorithm is shown below. See “Why entropy is expected to decrease as a consequence of regularity?” for an extended explanation of complexity and regularity. Our entire data set is conformed by 3D-matrices. It consists of n EEG. Each EEG has q channels by N data where each channel corresponds to one time series. Any EEG data set defined like this permits the computation of ApEn and the other entropies. Our ApEn algorithm is an adaptation from (Pincus 1991) taking advantage of three dimensional arrays in MATLAB (Zavala-Yoé 2008). Our remaining algorithms are based in such 3D matrices. 

ApEn computes the conditional probability of similarity between a data segment of a given duration and the next set of segments of the same duration. Thus, a lower value of ApEn means a high degree of regularity which indicates low complexity of data.

#### Sample entropy, SaEn

This statistic was defined in (Richman and Moorman 2000) in order to avoid some disadvantages ApEn has. For instance, although useful, ApEn is a biased statistic as a result of including *self matches*. This happens when the distance (defined in Algorithm 1) is measured for the *same vector x*, i.e., $d(x,x)=0$. In order to reduce such bias in the ApEn algorithm (step 8) the distance definition has to be modified to $d(x(i_{2},1,k_{2}),x(i_{2},1,k_{3}))=max_{i_{2},k_{2} \ne k_{3}}|x(i_{2},1,k_{2}),x(i_{2},1,k_{3})|$. Basically, the algorithm to compute SaEn and ApEn remains the same considering the latter constraint (Richman and Moorman 2000; Pincus 1991). 

The complexity plot for SaEn is given in Fig. 8. The sine function resulted to be the least complex signal among all and the white noise signal and the EEG-series the most complex. In the case of the chaotic system and the Brownian motion they are in the middle as result of their repetitive nature. This is revealed in the phase-space trajectories for the former and by self similarity patterns for the latter as a function of time (Stam et al. 1997; Mikosch 1998). The length of the series was 10,000 samples (equivalent to 50 s of EEG time). Recall that X7 behaves predominantly normal here.

#### Multiscale entropy, MSE

Computation of MSE is based on the definition of SaEn (Costa et al. 2002). As seen, from the algorithm to compute SaEn, the time scale is only one in the sense that a given time series (EEG channel) lasts from time $t_{1}$ to $t_{10000}$. SaEn is quite dependent on time series length, actually its standard deviation is also inversely proportional to the length of the time series (see definition of SaEn and ApEn above and Richman and Moorman 2000). This fact may cause that some times, ApEn and SaEn take a high value when it is calculated in certain pathologic time series that are assumed to represent less complex dynamics than to time series obtained from healthy patients. Instead of considering only one time scale, in (Costa et al. 2002) it is proposed to create finer time scales based in the original one. So, given a time series $X_{i}$ a coarse-grained time series $Y_{j}^{\tau }$ is constructed by taking pieces of $X_{i}$ of certain length $\tau$ and rescaling these pieces of $X_{i}$ in terms of this length (Costa et al. 2002). The algorithm to determine MSE was taken from (Costa et al. 2002) but we modified step 4 (see also note below): 

In this work, we propose to modify step 4 above as: MSE = SaEn($Y_{j}^{\tau }$) with $r(\tau )=0.2\sigma (Y_{j}^{\tau })$ i.e., considering r as a function rather than a constant. This continuous updating of r permits to compute a better variance of $Y_{j}^{\tau }$ because r is not calculated from $X_{i}$ as $r=0.2\sigma (X_{i})$ but from each sub series $Y_{j}^{\tau }$. This fact makes $\sigma$ inversely proportional to the length of each sub series $Y_{j}^{\tau }$ instead of keeping such inverse proportionality relative to *N*. The tolerance r is so being adjusted for each sub series $Y_{j}^{\tau }$. The behavior of complexity for the MSE case is shown in Fig. 9. Good results in modeling pathologies have been obtained by MSE (Costa et al. 2002).

#### Composite multiscale entropy, CMSE

An additional nonlinear metric to measure complexity is provided by the so called *composite multiscale entropy*, CMSE (Shuen-De 2013). Such metric constructs k coarsed -grained subseries from those already obtained by the MSE algorithm as $Y_{k}^{\tau }= Y_{k,1}^{\tau }, Y_{k,2}^{\tau },\ldots ,Y_{k,j_{max}}^{\tau }, j_{max}=N/\tau _{max}$. Next, SaEn is computed for each sub coarsed -grained subseries. CMSE is calculated as the result of averaging all the SaEn computed for each sub coarsed-grained time series for each value of $\tau$ (Algorithm 5.3.6). 

This figure is not included but we realized that the complexity pattern persists (with respect to SaEn and MSE but a little bit less to ApEn) but their plots look smoother (high frequency activity is missed) as a result of the extra coarse -graining process and by taking r (tolerance) as function of each subseries $Y_{k}^{\tau }= Y_{k,1}^{\tau }, Y_{k,2}^{\tau },...,Y_{k,j_{max}}^{\tau }$ (see step 4 in Algorithm 5.3.5 and steps 3 and step 4 in Algorithm 5.3.6). This also means that we have improved the original algorithm developed for CMSE (Shuen-De 2013). It is also noteworthy mention that the processing time used by the CMSE algorithm is much longer than that of ApEn, SaEn and MSE as a consequence of the extra coarse-graining processes plus the computation of SaEn for each of them (compare Algorithms 2, 3 and 4).

All the entropy measures could reveal the associated complexity of the test signals correctly. However, when they were assessed in the EEG database, our improved version of MSE ended up to be the best. ApEn and SaEn are variance sensible to the length of time series and CMSE, although improved here as well, needs much more computer memory than the other statistics. MSE did not show these problems (Zavala-Yoé et al. 2015).

### Description of comparisons: static and dynamic BMSE

**Fig. 17:**
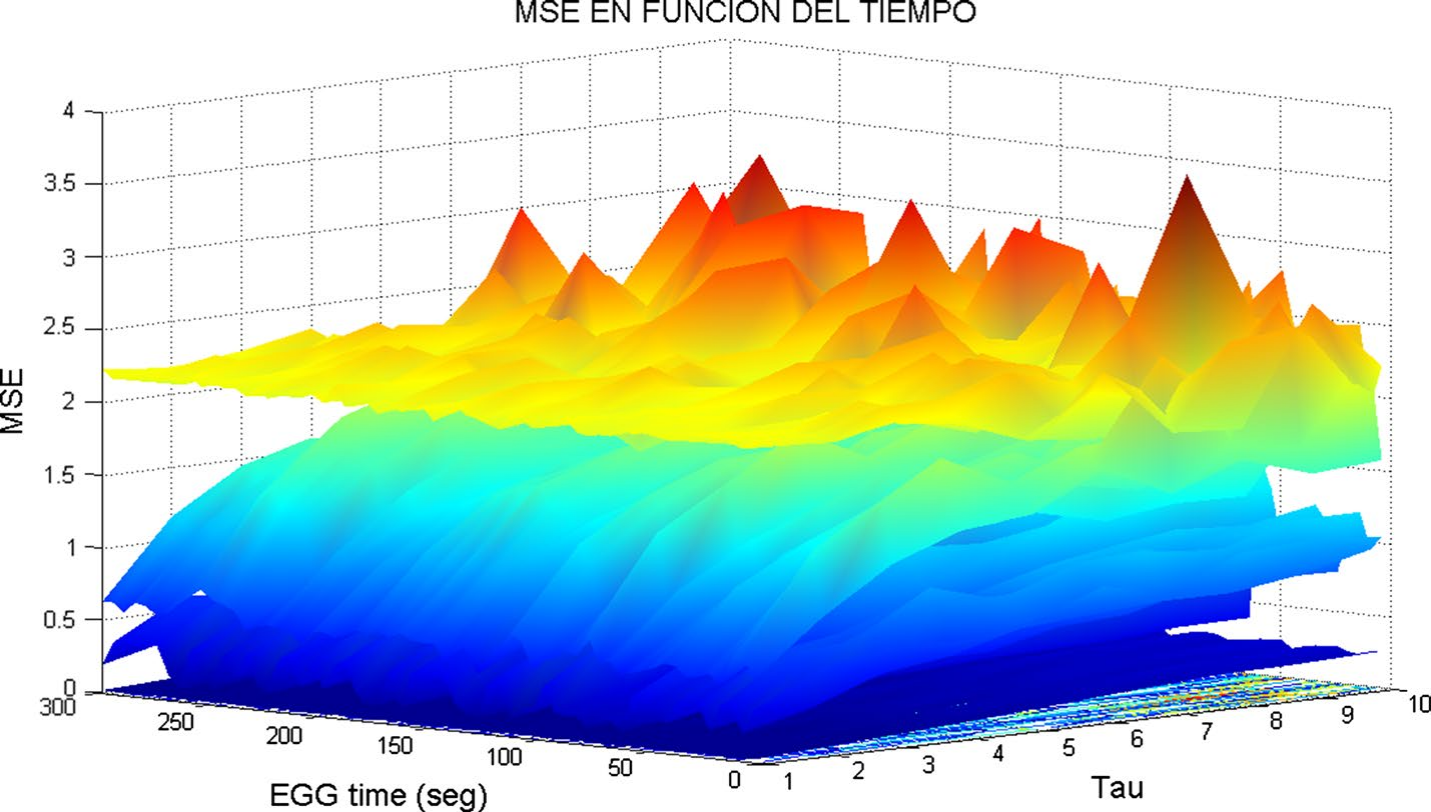
BMSE during 5 min time. The BMSE plotted at *bottom* corresponds to the sine wave and the highest corresponds to white noise. Compare with Fig. 9.



As a result of the succeed of MSE in “Results and discussion” it is proposed here to construct an enhanced version of such MSE, referred to as Bivariate MSE. This means that an MSE value will be computed for each time scale factor $\tau$ but during an arbitrary duration of the EEG channel, having in this way that BMSE is defined as $BMSE=MSE(t,\tau )$. This makes a big advantage over traditional MSE and EEG because for the former there will be a tree dimensional plot which will comprise information in only one surface. For the latter, the evolution of this affection can be studied better as it will be explained in “Results and discussion”. An interesting 3D plot can be generated by plotting $\tau$, the time scale factor, the time axis t, and MSE computed for an arbitrary interval of time $\Delta t$. This can be done by placing together complexity MSE-plots corresponding to continuous periods of time (slices, see Algorithm 5). For instance, the MSE-plot corresponding to the first 10 seconds is placed next to the MSE-plot corresponding to the following 10 seconds and so on. All together will form a three dimensional surface considering (in this example) n times 10 seconds. Of course the period of time can be anyone. Thus, we can bring together a general 3D interpretation of MSE complexity. Compare now MSE in Fig. 9 and BMSE in Fig. 17. The complexity pattern persists for the all signals. In all the BMSE plots, the BMSE variations are chromatically shown by gradual alterations of colors which go from dark blue to red. Dark blue means low complexity, i.e., $BMSE\le 1$. The value for BMSE = 1 is indicated by a lighter tone of blue. Relative high values of complexity, i.e., $1<BMSE<1.5$ are colored in tones which vary slowly from light blue to green and yellow, phase by phase. Higher complexities are two toned in orange and red. The algorithm which computes and plots these complexity surfaces is given below.

In step 7 of Algorithm 5.4, ”Surface” means to construct a 3D structure to plot a 3D MSE graph properly. In addition, 3D-arrays were used to simplify computations (Zavala-Yoé 2008). The complexity pattern coincides with the univariate version of MSE. Note that in this case, as lower the curve (for $\tau =1$ and so on) as less complex the signal. The MSE surface at the bottom corresponds to the sine wave. The one which appears at top, corresponds to white noise. Those in the middle are the MSE surfaces of: Brownian motion, one state variable, $x_{1}$, of the chaotic system and one EEG signal (X7), respectively. Once with this algorithm to compute the BMSE, it is also possible to create a 3D animation for a set of BMSE surfaces as follows. This algorithm will be used in the following section.

### Numerical experiments

The content of the Methods section converges to the following list of numerical experiments. Compare this part with “Contributions as goals” and “Results and discussion”.

ApEn, SaEn, MSE and CMSE were used in part 1 below. MSE was used from 2-4 and BMSE from 5-10.

1. Assessing of four entropies to obtain only one which can be used to study the evolution of this affection based in massive data (MSE succeeded).
2. Interpreting and comparing the three states of a seizure in traditional voltage-time plots with their respective complexity (MSE) plots.
3. The chosen entropy (MSE) will be applied in multiple long term EEG to give simultaneous long term information during the three states of a seizure.
4. The chosen entropy (MSE) will be used to find which region of the brain behaves worst and on which year.
5. From the aforementioned entropy, a new one will be designed (BMSE) to exhibit much longer term information by means of 3D surfaces and 3D animations (SBMSE and DBMSE, repectively).
6. Analysis of 90 seconds time containing multiple preictal, ictal and postictal phases by BMSE.
7. Comparison of BMSE and MSE in X19 of EEG2 (2010).
8. Description of long term BMSE complexity in 2.5 hours time of channel X1 in EEG4.
9. Comparison among X17, X18, X19 and X20 in EEG4 during 10 minutes.
10. DBMSE: Computational animation of 2.5 h of BMSE evolution in EEG4.

Recall that, in a EEG, a preictal stage is characterized by an asynchronous pattern with relative low voltage amplitude. As a consequence, the complexity is higher in this case than during an ictal phase where the neurons are synchronized exhibiting high voltage amplitude peaks. Hence, the complexity comes down. During the postictal period, the voltage amplitude starts to decrease slowly increasing complexity gradually. These characteristics were well reflected by BMSE (see “Results and discussion”).

## Why entropy is expected to decrease as a consequence of regularity?

In “Aproximate entropy, ApEn”, the concept of ApEn was explained and it also was emphasized that as a result of some drawbacks, SaEn replaced ApEn (“Sample entropy, SaEn”). Our evaluation among ApEn, SaEn, MSE and CMSE revealed that our modified version of the original MSE was the best statistic to be applied to our EEG-database. Since our MSE is based in the calculation of SaEn, with no loss of generality, this section is written in terms of SaEn. In “Aproximate entropy, ApEn”, “Sample entropy, SaEn”, “Multiscale entropy, MSE”, and “Composite multiscale entropy, CMSE” it is explained that given a time series X, such X is arranged in a collection of $x(i), i=1,\ldots ,N-m+1$ sub vectors (*templates* (Pincus 1991; Richman and Moorman 2000) of length *m*. SaEn counts the number of these vectors *x*(*i*) whose component-wise distance *d* to each other is lower than a tolerance defined as $r=0.2\sigma (X)$. As more sub-vectors satisfy this constraint, as more regular X is (Pincus 1991; Richman and Moorman 2000; Costa et al. 2002; Shuen-De 2013; Chon et al. 2009). Why? Well, first, recall that, in “Aproximate entropy, ApEn”, the Kolmogorov-Smirnov entropy was defined by means of Eq. 1. This equation is approximated as follows:

5 $$\begin{aligned} KS_{Entropy}=\lim _{r \rightarrow 0} \lim _{m \rightarrow \infty } \lim _{N \rightarrow \infty }\left[\Phi ^{m}(r)-\Phi ^{m+1}(r) \right]\approx \Phi ^{m}(r)-\Phi ^{m+1}(r) \end{aligned}$$

In Pincus (1991) ApEn is defined as

6 $$\begin{aligned} ApEn=\Phi ^{m}(r)-\Phi ^{m+1}(r)=\frac{1}{N-m+1}\sum _{i=1}^{N-m+1}\ln \Bigg (\frac{C_{i}^m(r)}{C_{i}^{m+1}(r)} \Bigg ) \end{aligned}$$

The argument of the logarithm is a probability of occurrence of such *x* satisfying the above mentioned distance tolerance for sub-vectors *x* of length *m* and $m+1$, denoted as $\sharp x(i)|_{m,d \le r}$ and $\sharp x(i)|_{m+1,d \le r}$, respectively.

7 $$\begin{aligned} P(i)=\frac{C_{i}^m(r)}{C_{i}^{m+1}(r)}=\frac{\sharp x(i) \big |_{m,d \le r}}{\sharp x(i) \big |_{m+1,d \le r}} \end{aligned}$$

The component-wise distance constraint $d(X(\tau ),X(\tau \Delta \tau ))=|X(\tau )-X(\tau \Delta \tau )| \le 0.2 \sigma (X(\tau ))$, $\tau =[\tau _{min}:\Delta \tau :\tau _{max}]$, $\Delta \tau =1,2,\ldots ,\Delta \tau _{max}$, $\Delta \tau _{max}=\tau _{max}-1$, $\tau _{max}=N-m+1$, can be written as $d(X(\tau ),X(\tau \Delta \tau ))/\sigma (X(\tau ))\le s, 0\le s \le 1$ for an arbitrarily value of *s*. The latter ratio measures the change in amplitudes with respect to the signal variation. In (Pincus 1991; Chon et al. 2009; Costa et al. 2002; Richman and Moorman 2000) values of $s=0.15, 0.2$ are considered either by definition or as a result of experiments. Hence, a sampled signal is considered as regular if that ratio is less than *s*; we also considered $s=0.2$.

Consider now the definition of SaEn (“Sample entropy, SaEn” and Richman and Moorman 2000). Roughly speaking, SaEn looks the same as Eq. 6 but considering the distance constraint as $d(X(\tau ),X(\tau \Delta \tau ))=|X(\tau )-X(\tau \Delta \tau )| \big |_{\Delta \tau \ne 1} \le 0.2 \sigma (X(\tau ))$. With this clarification, we can study the complexity of a periodic sampled signal *X* in terms of SaEn. Since a periodic signal repeats its pattern every *n* periods *T* for $n=1,2,3,\ldots$, SaEn can be expressed in this case as

8 $$\begin{aligned} SaEn=\frac{1}{N-m+1} \sum _{j=1}^{n} \sum _{i=1}^{\frac{N-m+1}{nT}}\ln \Bigg (\frac{C_{i}^m(r)}{C_{i}^{m+1}(r)} \Bigg ) \end{aligned}$$

The signal will be the same for all periods *nT*, so it is possible to write Eq. 8 as follows:

9 $$\begin{aligned} SaEn=\frac{n}{N-m+1} \sum _{i=1}^{\frac{N-m+1}{T}}\ln \Bigg (\frac{\sharp x(i) \big |_{m,d \le r}}{\sharp x(i) \big |_{m+1,d}} \Bigg )=\frac{n}{N-m+1}S \end{aligned}$$

Where *S* denotes the summation. If a signal is periodic or preponderantly periodic (as in the case of an epileptic seizure) then it follows that $\sharp x(i) \big |_{m,d \le r} \approx \sharp x(i) \big |_{m+1,d \le r}$. This implies that $P(i) \approx 1$ for many sub-vectors *x* and in this case, $ln(P(i)) \approx 0$, and hence $SaEn \approx 0$, i.e., low complexity. So, small values of SaEn mean low complexity. Moreover, if we assume that $\frac{n}{N-m+1}S>1$ then $S>\frac{N-m+1}{n}$. However, the number of samples *N* of *X* is of order of magnitude 4,5 or even 6 for EEG records (which last from some minutes to about 2 hours) while *S* will be very small because $ln(P(i)) \approx$0. It follows that the latter inequality is false and $SaEn<1$ for periodic and approximately periodic signals. Analogously, for a random signal where -in contrast- $\sharp x(i) \big |_{m,d \le r} \approx 0$ then $P(i) \approx 0$ and $ln(P(i)) > 1$, and $SaEn > 1$, i.e., high complexity.

Now, consider that $SaEn=1$. Let us write Eq. 6 for *SaEn* as $SaEn=(N-m+1)^{-1}(s_{1}+ \cdots +s_{N-m+1})$. As all the terms of the summation are positive, they have to be less or equal than one. If at least one $s_{i}=1$, then $s_{j}=0, i \ne j, i=1,2,\ldots ,N-m+1$. In this case, all the complexity of the signal ($SaEn=1$) will only be equivalent to the i-th element complexity. This means that the relevant information is contained in that part of the signal and the other part will be periodic. Next assume that for any two terms $s_{i}, s_{j}, s_{i}+s_{j}=1, i \ne j, i=1,2,\ldots ,N-m+1$. This again means that the complexity of the complete signal is concentrated in two parts of the time series and again the signal behaves preponderantly periodic. When $s_{i}=(N-m+1)^{-1}, i=1,2,\ldots ,N-m+1$ the complexity of the whole signal is equally distributed through it in very tiny values of *SaEn*.

Since our MSE is also based on SaEn, the conclusions about complexity for our MSE are similar.

## Abbreviations

IMI: imipramine
PA: piracetam
VPA+: valproate sodium
TPM: topiramate
CLB: clobazam
VPA: valproic acid
LTG: lamotrigina
LEV: levetiracetam
ATX: atomoxetine
ESM: ethosuximide
PSE: prednisone
MDZ: midazolam
CZP: clonazepam
ZNS: zomisamide
CZP: clorasepate dipotassium
PB: phenobarbital
LCM: lacosamide
PRM: primidone
Q10: co-enzime Q10 (not an anticonvulsant).
Letters *a* and *b*: first and second semester of 2013, respectively.
